# Supplementary material for: Synthesis of Brominated Lactones Related to Mycalin A: Selective Antiproliferative Activity on Metastatic Melanoma Cells and Inhibition of the Cell Migration
Source: Mar Drugs. 2023 Jun 7;21(6):349. doi: 10.3390/md21060349 (PMC10301594; doi:10.3390/md21060349)
Supplement: Supplementary file 1 [file marinedrugs-21-00349-s001.zip › marinedrugs-2363085-supplementary.pdf]

Supplementary Information

Synthesis of Brominated Lactones related to Mycalin A. Selective Antiproliferative Activity on Metastatic Melanoma Cells and Inhibition of the Cell Migration

CONTENTS:

Figure S1: Comparative analysis of antiproliferative activity of alcohols **5–7**; **10–12**, **19**, and **21** on HeLa, A375, and WM266 tumour cells.

Figure S2: ^{1}H NMR spectrum of compound **4** ($CDCl_3$, 500 MHz).
Figure S3: ^{13}C NMR spectrum of compound **4** ($CDCl_3$, 175 MHz).

Figure S4: ^{1}H NMR spectrum of compound **5** ($CDCl_3$, 500 MHz).
Figure S5: ^{13}C NMR spectrum of compound **5** ($CDCl_3$, 125 MHz).

Figure S6: ^{1}H NMR spectrum of compound **6** ($CDCl_3$, 500 MHz).
Figure S7: ^{13}C NMR spectrum of compound **6** ($CDCl_3$, 125 MHz).

Figure S8: ^{1}H NMR spectrum of compound **7** ($CDCl_3$, 500 MHz).
Figure S9: ^{13}C NMR spectrum of compound **7** ($CDCl_3$, 125 MHz).

Figure S10: ^{1}H NMR spectrum of compound **8** ($CDCl_3$, 500 MHz).
Figure S11: ^{13}C NMR spectrum of compound **8** ($CDCl_3$, 125 MHz).

Figure S12: ^{1}H NMR spectrum of compound **9** ($CDCl_3$, 500 MHz).
Figure S13: ^{13}C NMR spectrum of compound **9** ($CDCl_3$, 125 MHz).

Figure S14: ^{1}H NMR spectrum of compound **10** ($CDCl_3$, 500 MHz).
Figure S15: ^{13}C NMR spectrum of compound **10** ($CDCl_3$, 150 MHz).

Figure S16: ^{1}H NMR spectrum of compound **11** ($CDCl_3$, 700 MHz).
Figure S17: ^{13}C NMR spectrum of compound **11** ($CDCl_3$, 175 MHz).

Figure S18: ^{1}H NMR spectrum of compound **12** ($CDCl_3$, 500 MHz).
Figure S19: ^{13}C NMR spectrum of compound **12** ($CDCl_3$, 125 MHz).

Figure S20: ^{1}H NMR spectrum of compound **13** ($CDCl_3$, 700 MHz).
Figure S21: ^{13}C NMR spectrum of compound **13** ($CDCl_3$, 175 MHz).

Figure S22: ^{1}H NMR spectrum of compound **14** ($CDCl_3$, 700 MHz).
Figure S23: ^{13}C NMR spectrum of compound **14** ($CDCl_3$, 175 MHz).

Figure S24: ^{1}H NMR spectrum of compound **15** ($CDCl_3$, 500 MHz).
Figure S25: ^{13}C NMR spectrum of compound **15** (CDCl3, 125 MHz).

Figure S26: ^{1}H NMR spectrum of compound **16** ($CDCl_3$, 500 MHz).
Figure S27: ^{13}C NMR spectrum of compound **16** ($CDCl_3$, 125 MHz).

Figure S28: ^{1}H NMR spectrum of compound **17** ($CDCl_3$, 500 MHz).
Figure S29: ^{13}C NMR spectrum of compound **17** ($CDCl_3$, 125 MHz).

Figure S30: ^{1}H NMR spectrum of compound **18** ($CDCl_3$, 500 MHz).
Figure S31: ^{13}C NMR spectrum of compound **18** ($CDCl_3$, 125 MHz).

Figure S32: ^{1}H NMR spectrum of compound **19** ($CDCl_3$, 500 MHz).
Figure S33: ^{13}C NMR spectrum of compound **19** ($CDCl_3$, 150 MHz).

Figure S34: ^{1}H NMR spectrum of compound **20** ($CDCl_3$, 600 MHz).
Figure S35: ^{13}C NMR spectrum of compound **20** ($CDCl_3$, 150 MHz).

Figure S36: ^{1}H NMR spectrum of compound **21** ($CDCl_3$, 700 MHz).
Figure S37: ^{13}C NMR spectrum of compound **21** ($CDCl_3$, 175 MHz).

Figure S38: ^{1}H NMR spectrum of compound **22** ($CDCl_3$, 700 MHz).
Figure S39: ^{13}C NMR spectrum of compound **22** ($CDCl_3$, 175 MHz).

HeLa cells

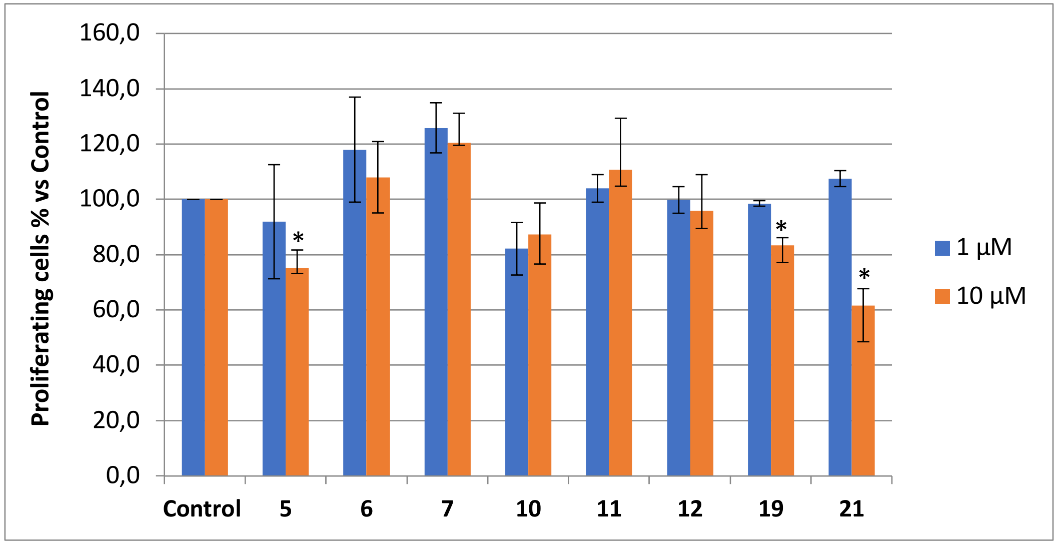

A375 cells

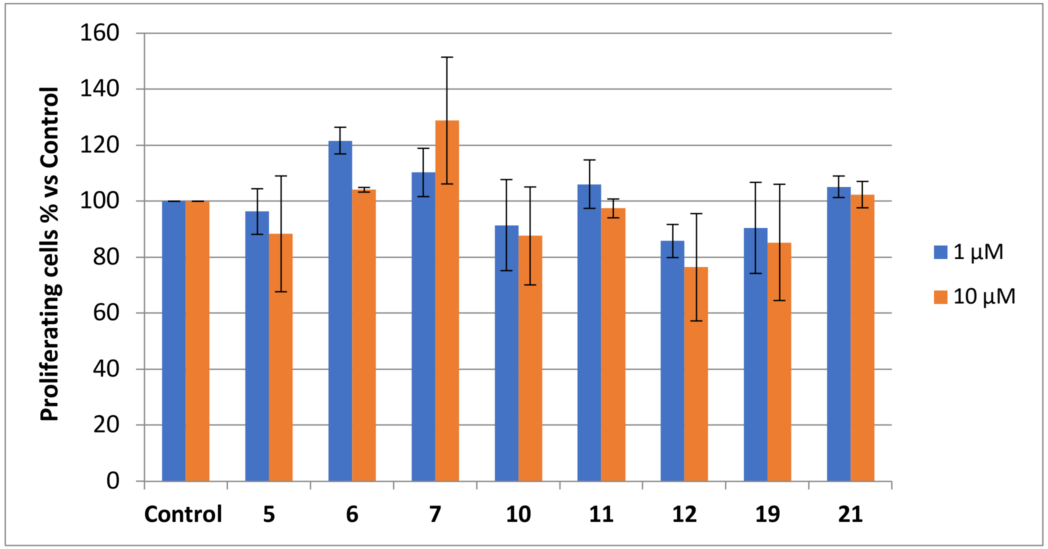

WM266 cells

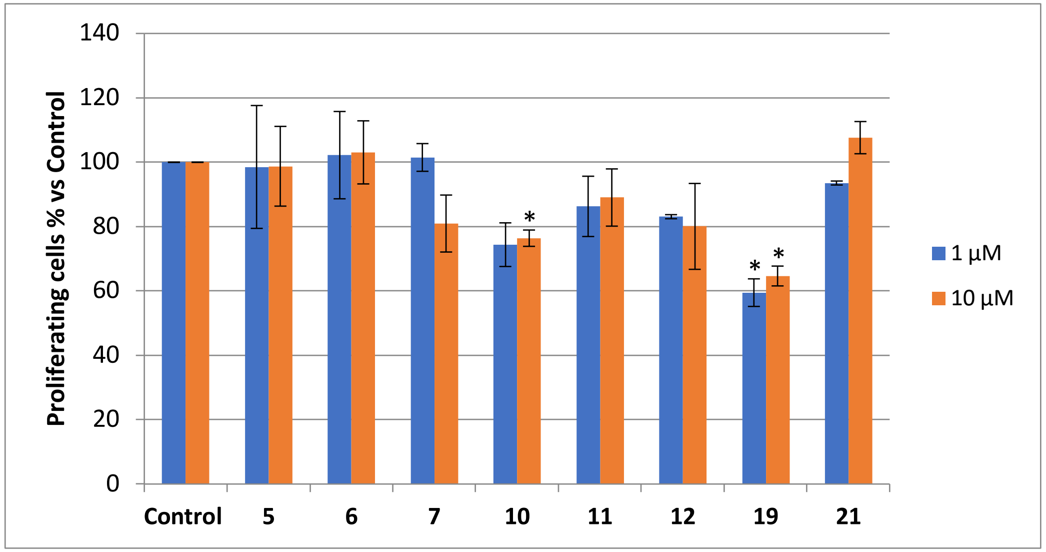

Figure S1. Comparative analysis of antiproliferative activity of all alcohols **5-7**; **10-12**; **19** and **21** on HeLa, A375, WM266 tumour cells. The cells were incubated at 37 °C, for 48 h in the presence of the substances. The proliferation was determined by MTT assays. The results are presented as the percentage of proliferating cells with respect to the control (vehicle treated cells) and are expressed as means ± SD of at least three independent experiments, * $p < 0.05$.

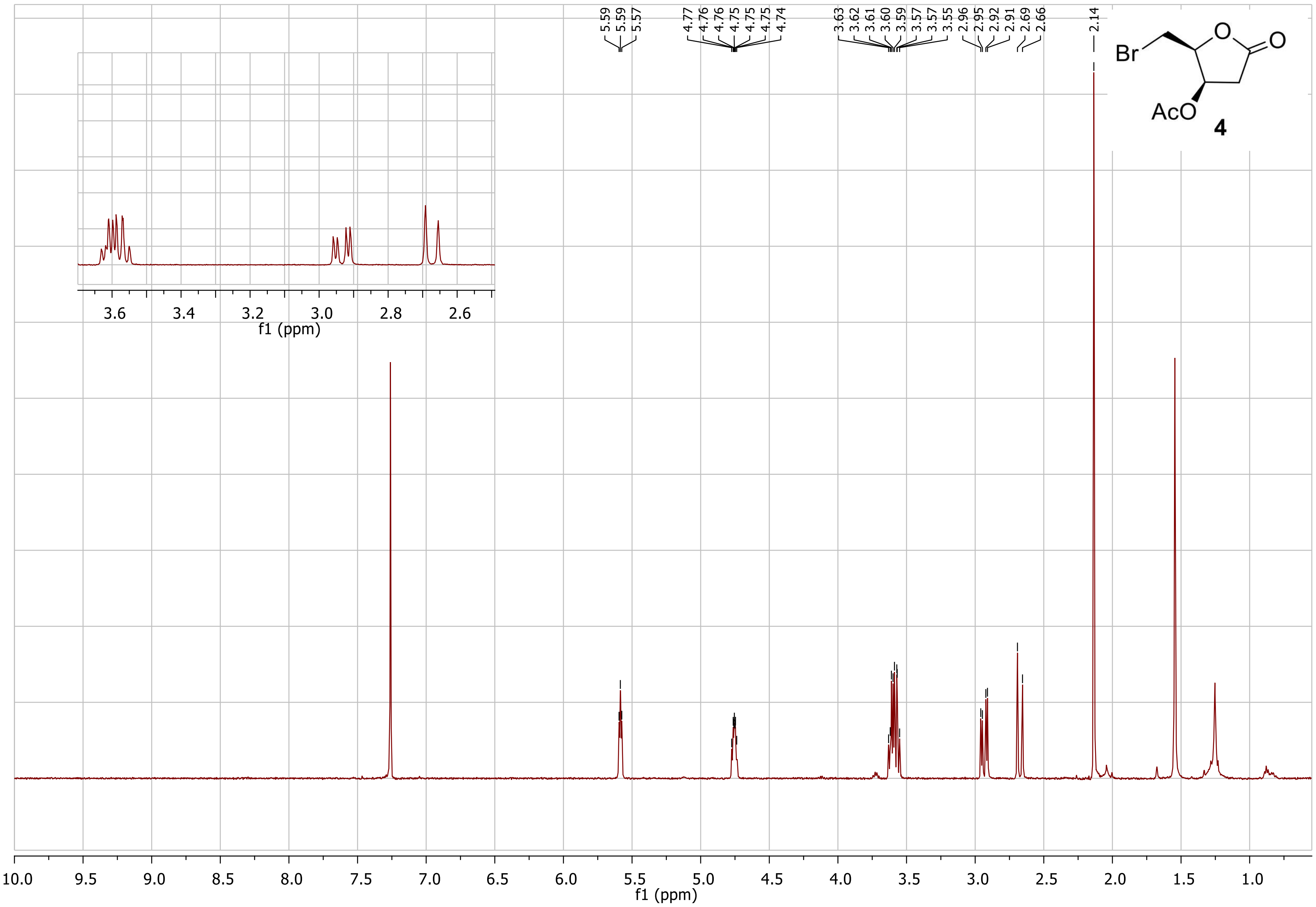

Figure S2: ^{1}H NMR spectrum of compound **4** ($CDCl_3$, 500 MHz).

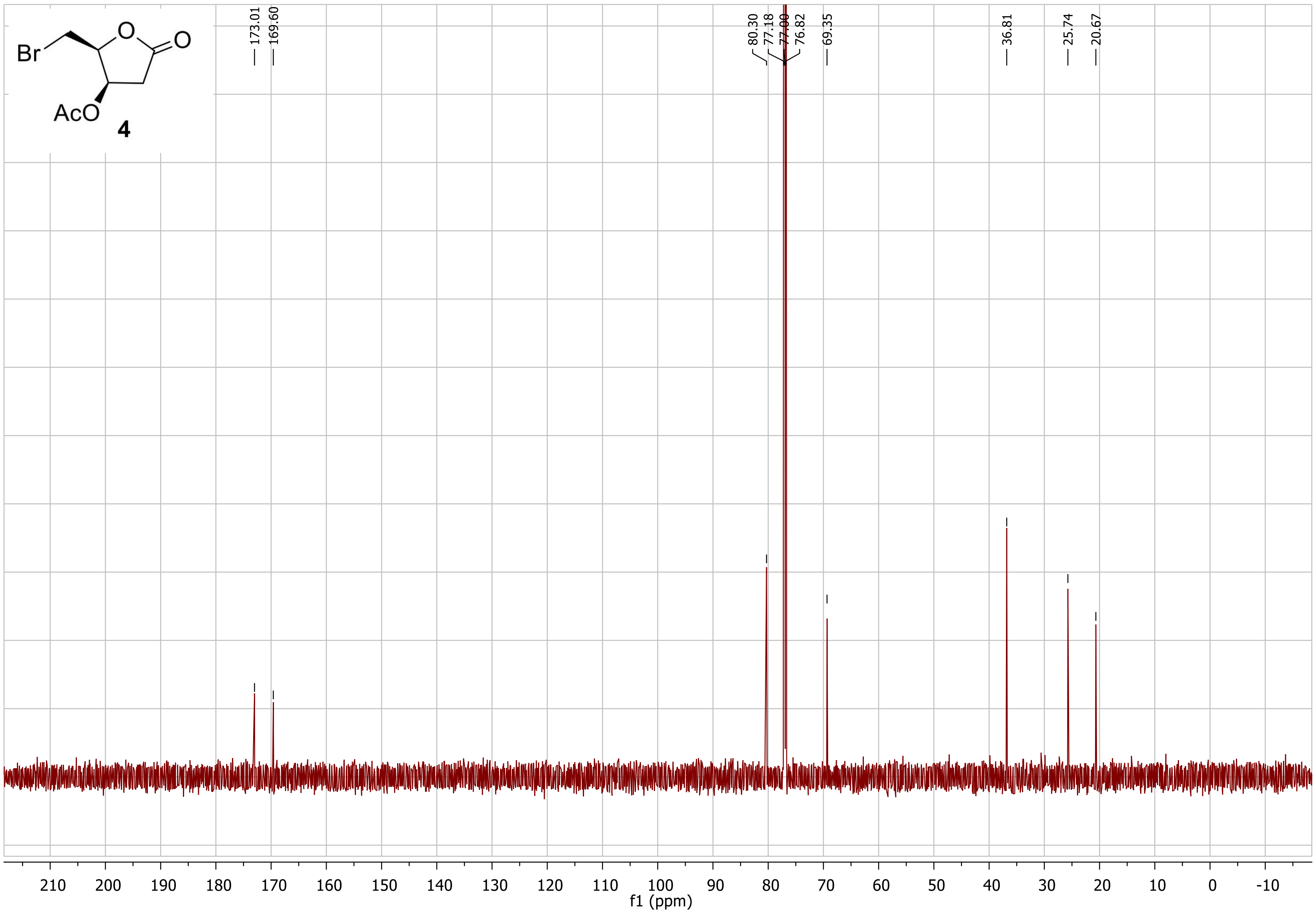

Figure S3: ^{13}C NMR spectrum of compound **4** ($CDCl_3$, 175 MHz).

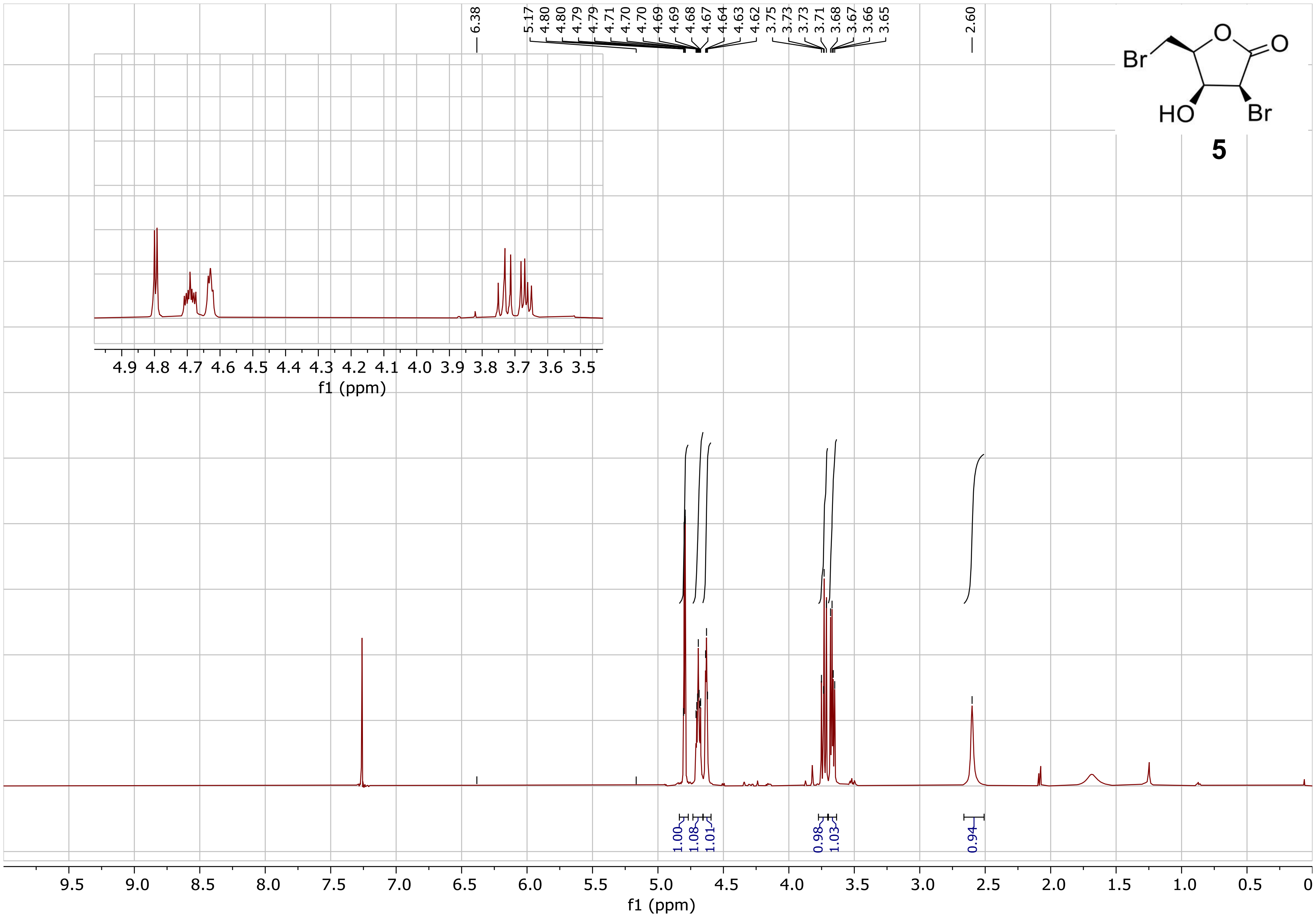

Figure S4: ^{1}H NMR spectrum of compound **5** ($CDCl_3$, 500 MHz).

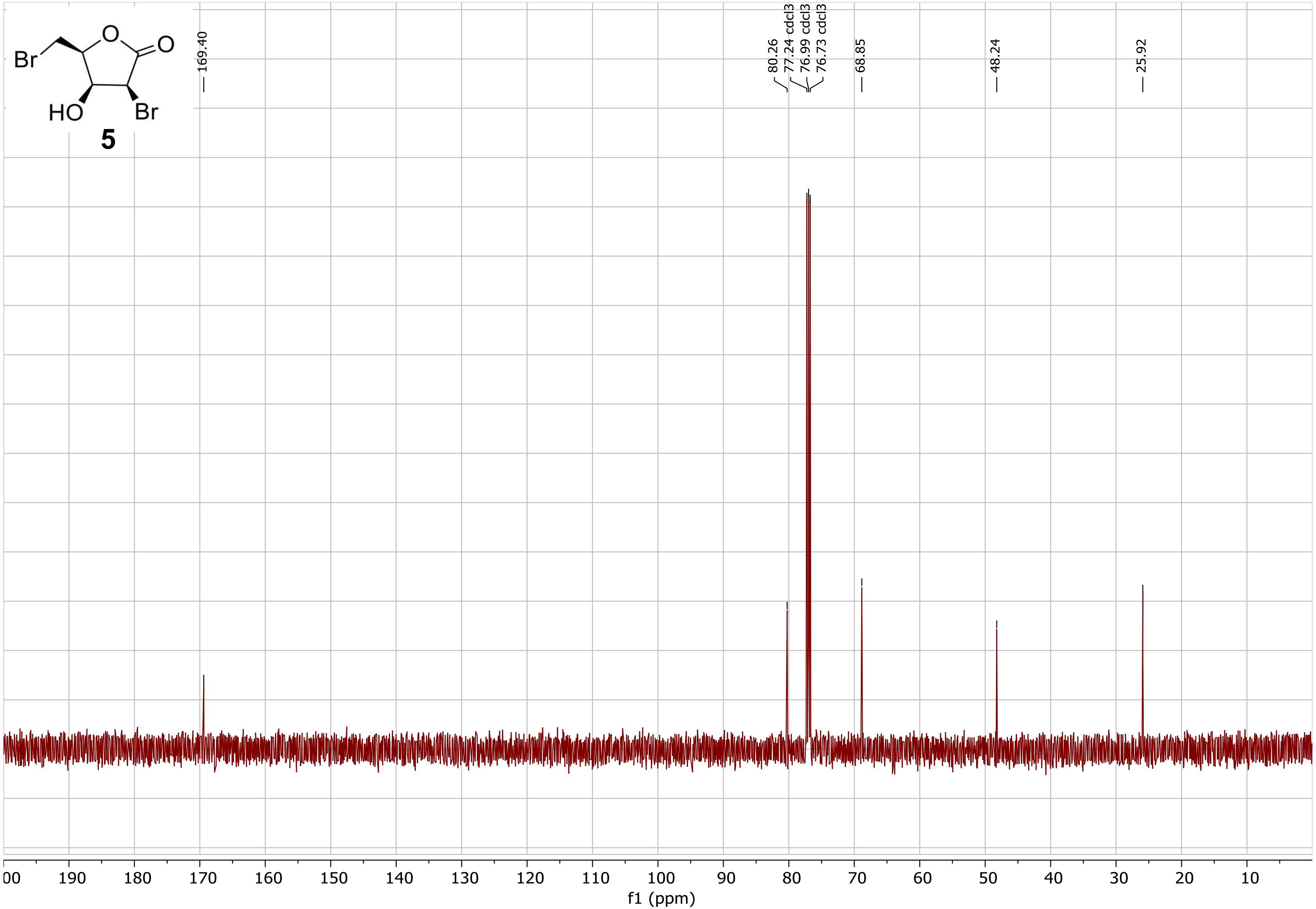

Figure S5: ^{13}C NMR spectrum of compound **5** ($CDCl_3$, 125 MHz).

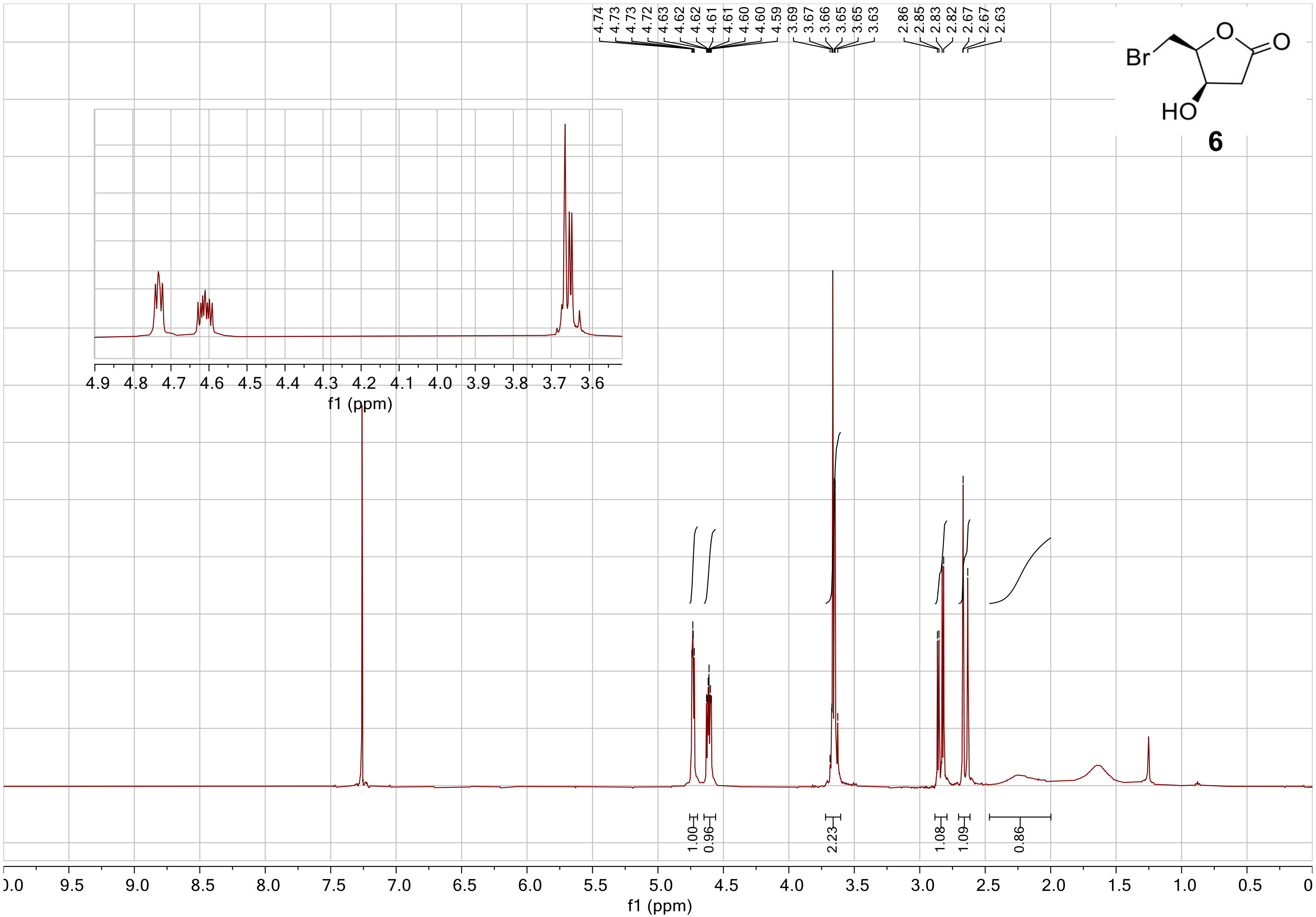

Figure S6: ^{1}H NMR spectrum of compound **6** ($CDCl_3$, 500 MHz).

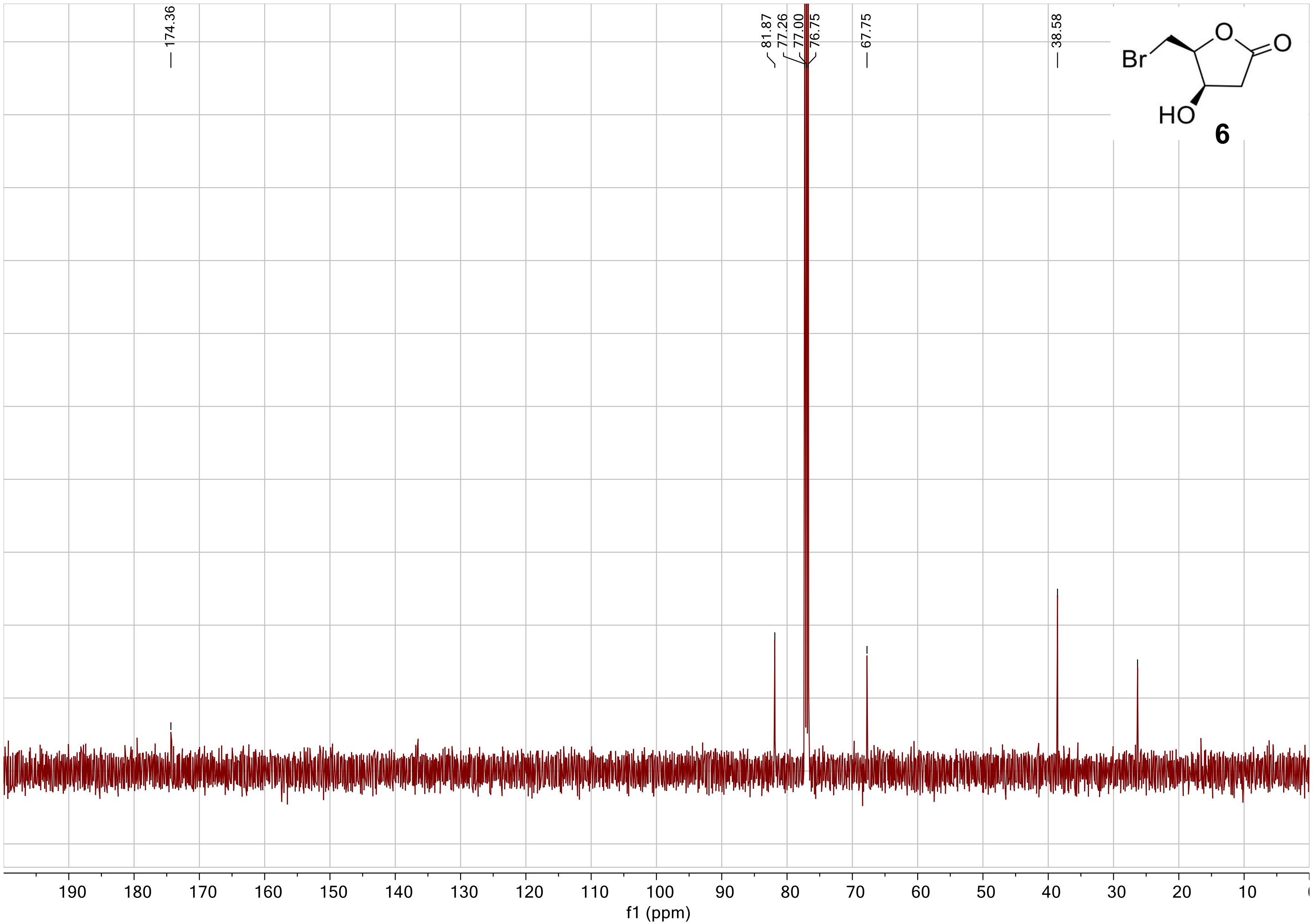

Figure S7: ^{13}C NMR spectrum of compound **6** ($CDCl_3$, 125 MHz).

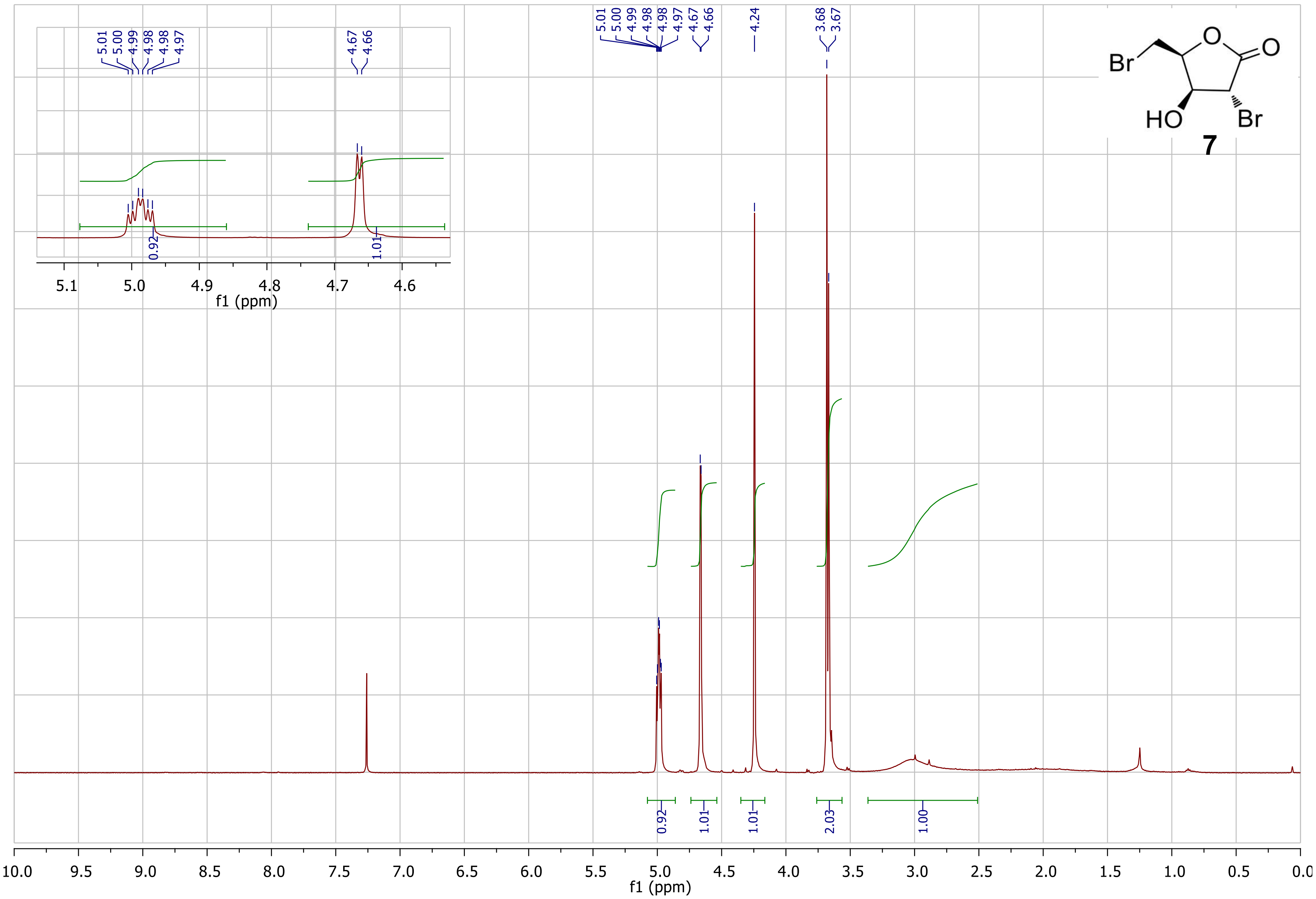

Figure S8: ^{1}H NMR spectrum of compound **7** ($CDCl_3$, 500 MHz).

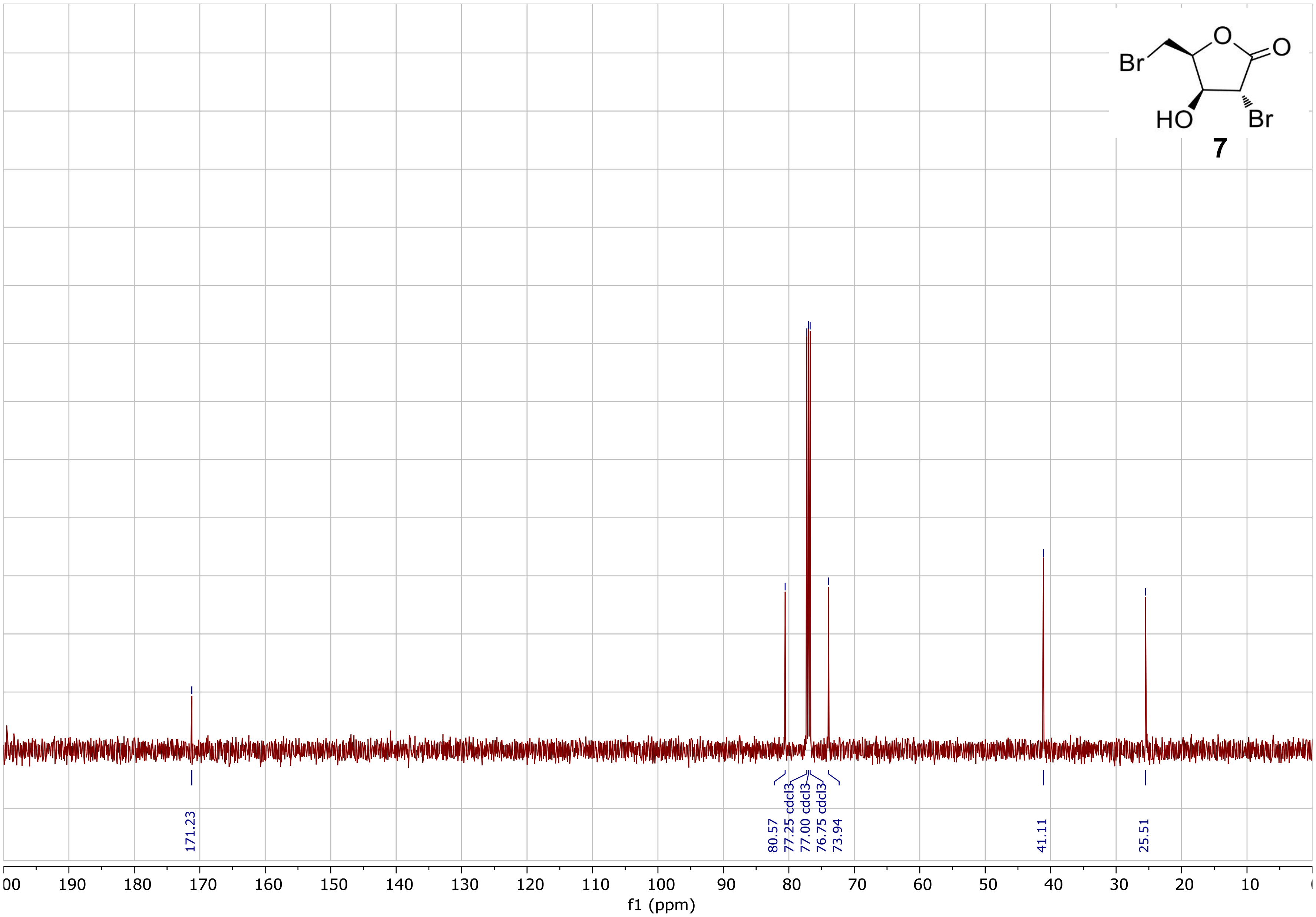

Figure S9: ^{13}C NMR spectrum of compound **7** ($CDCl_3$, 125 MHz).

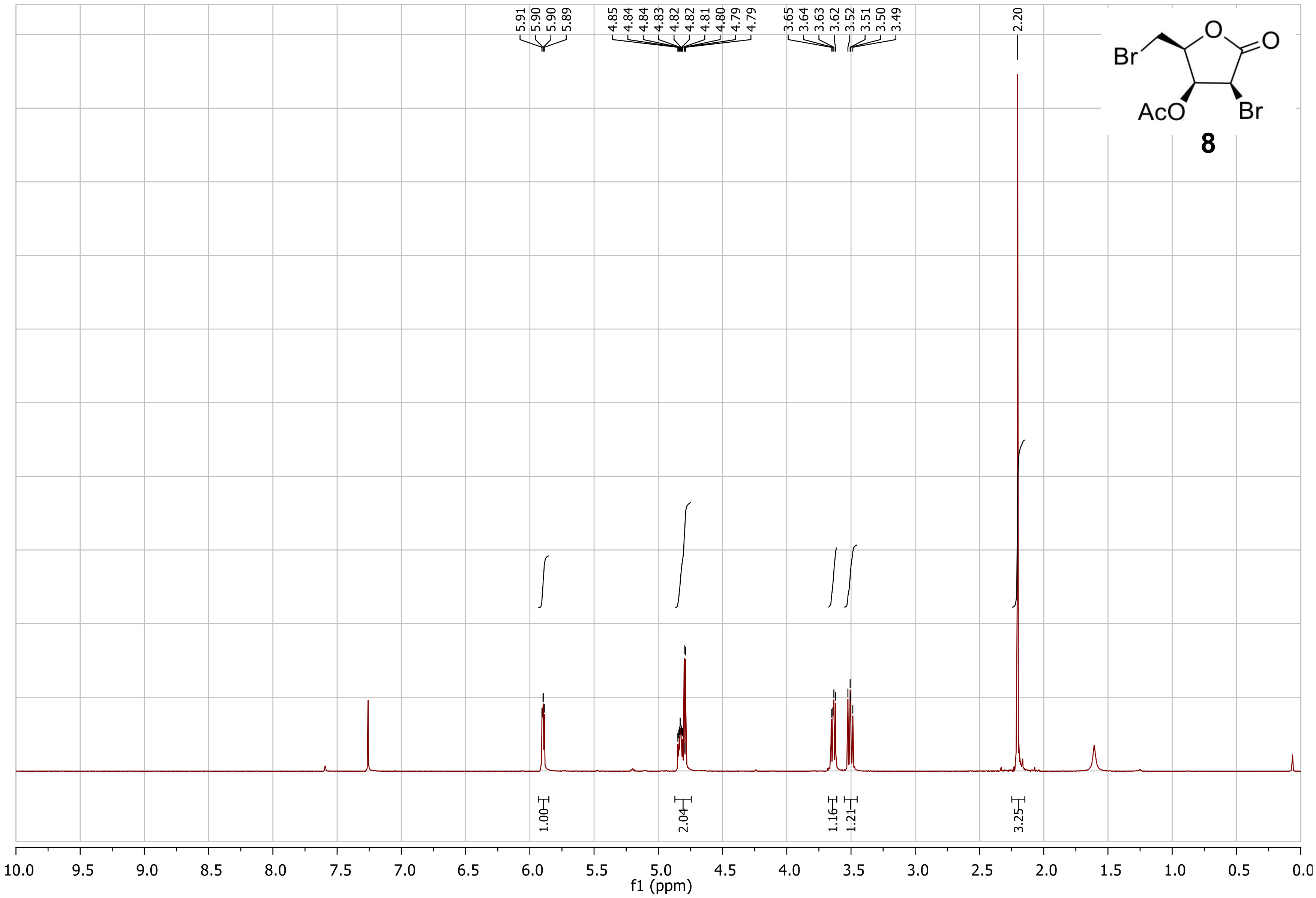

Figure S10: ^{1}H NMR spectrum of compound **8** ($CDCl_3$, 500 MHz).

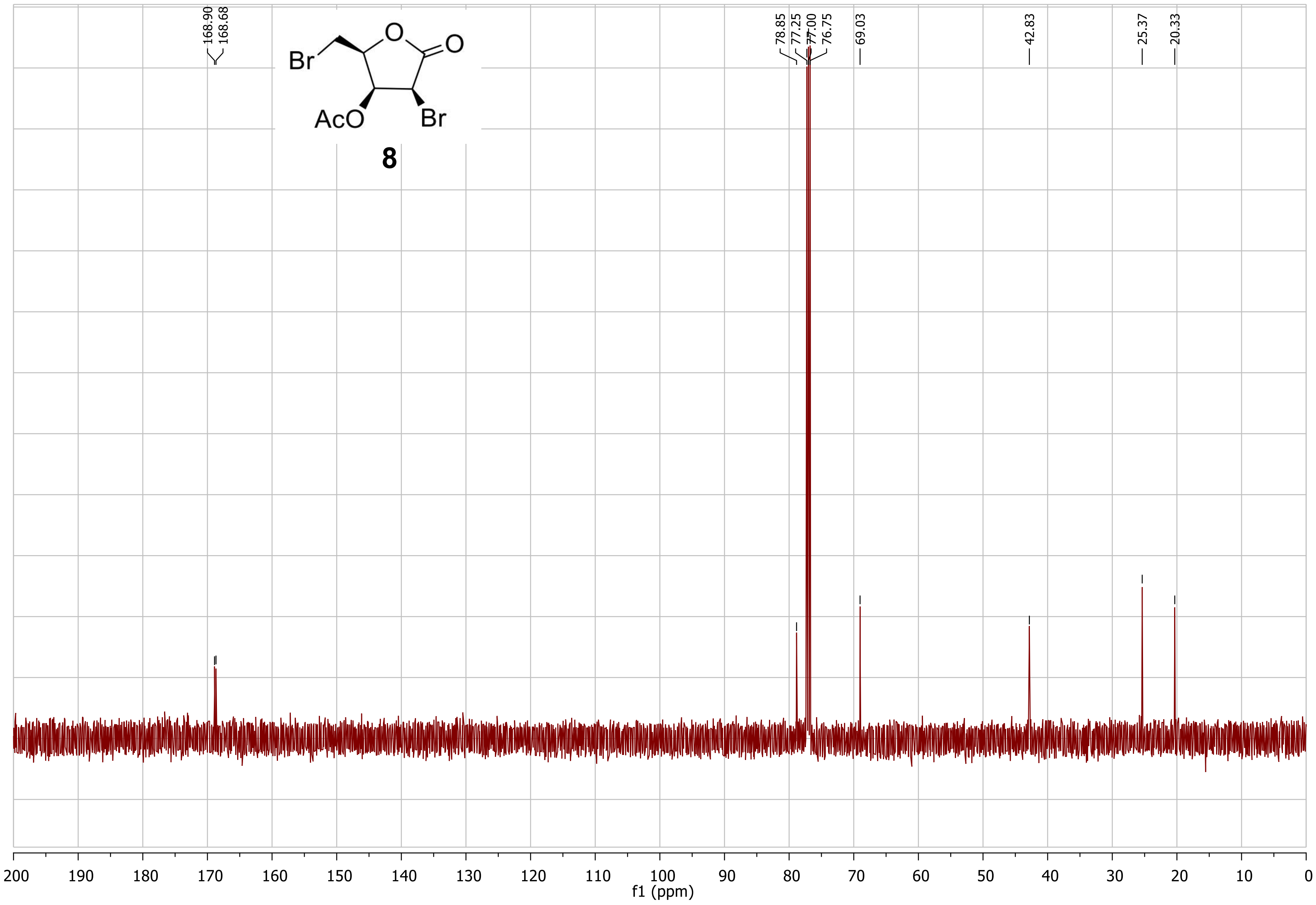

Figure S11: ^{13}C NMR spectrum of compound **8** ($CDCl_3$, 125 MHz).

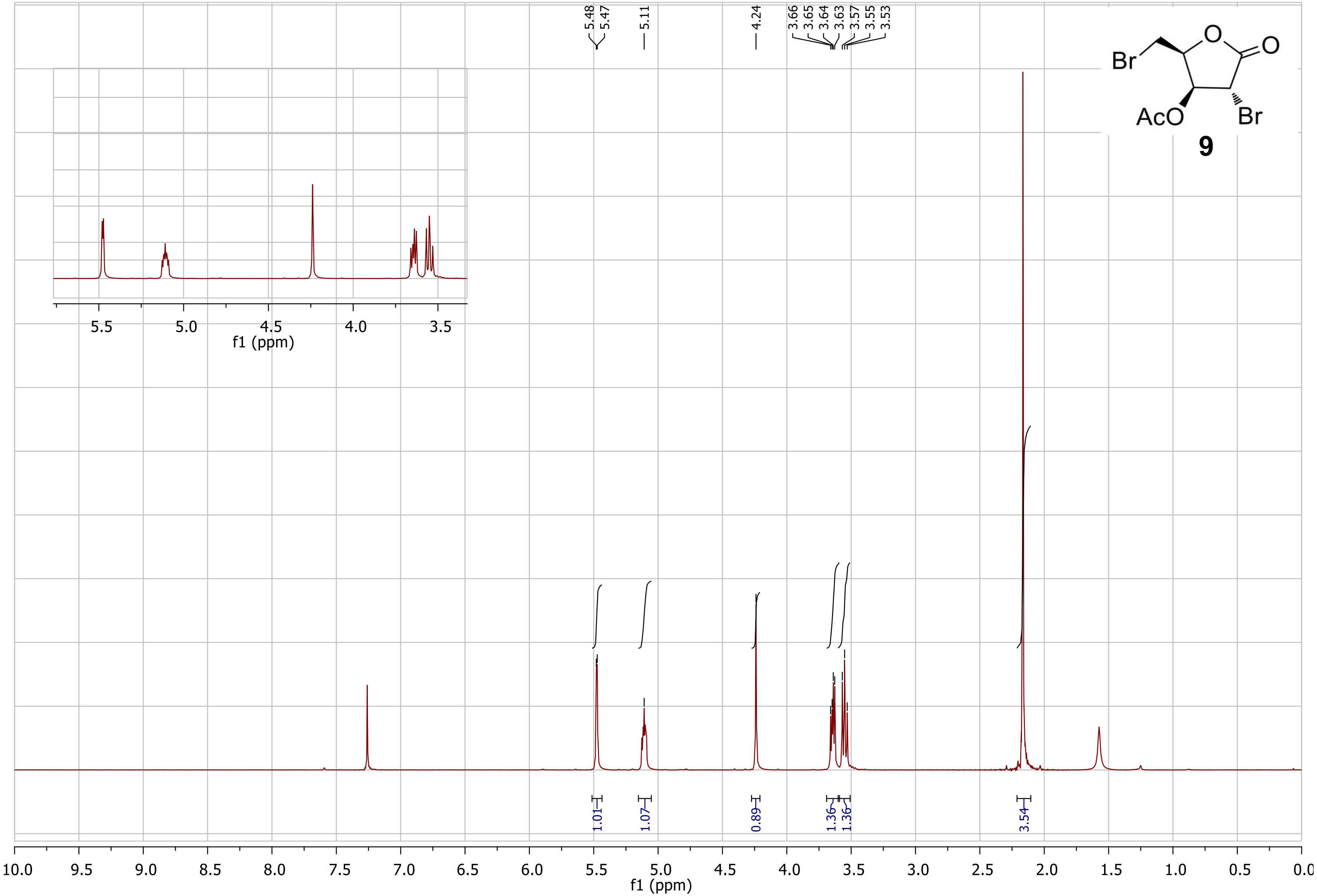

Figure S12: ^{1}H NMR spectrum of compound **9** ($CDCl_3$, 500 MHz).

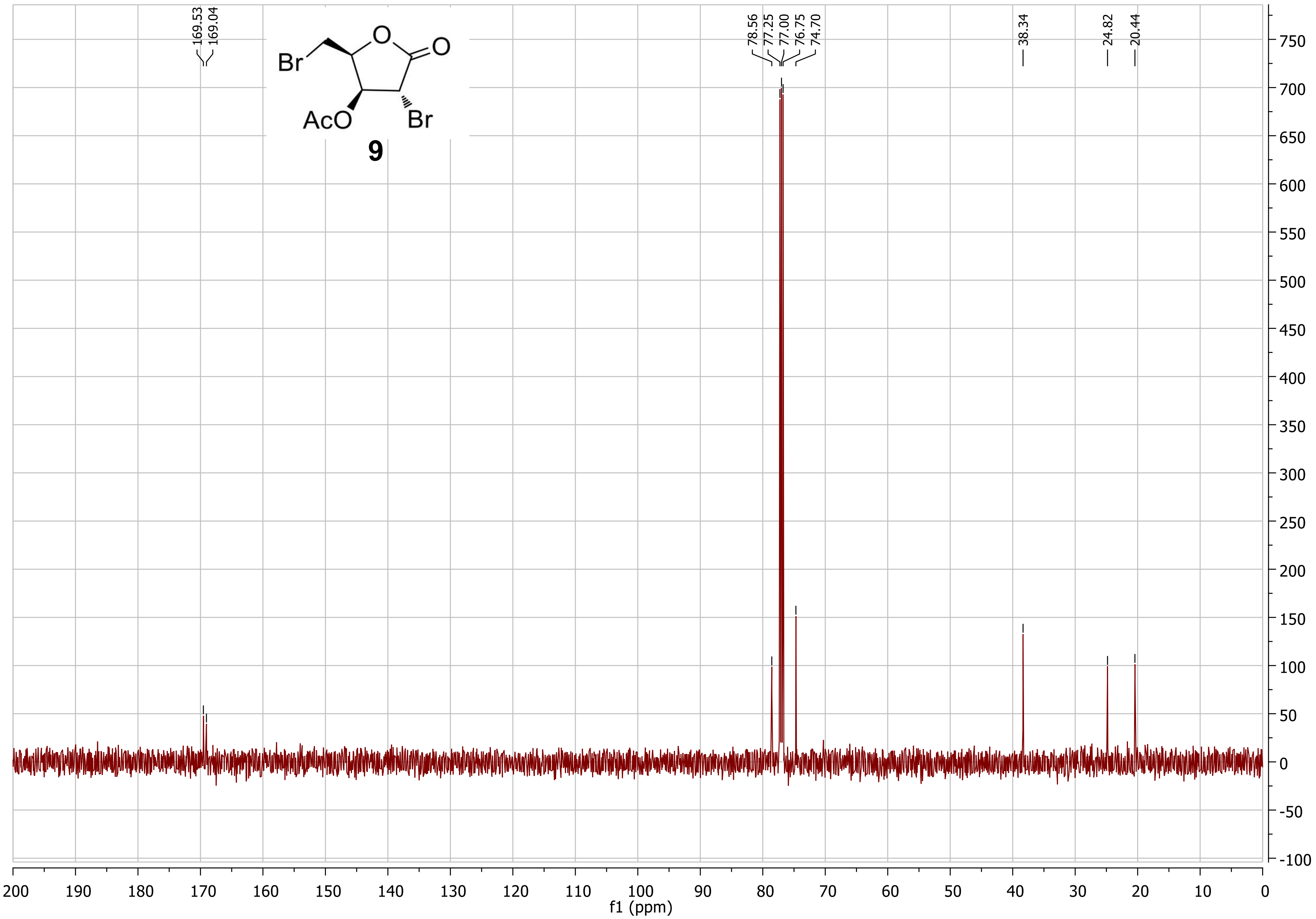

Figure S13: ^{13}C NMR spectrum of compound **9** ($CDCl_3$, 125 MHz).

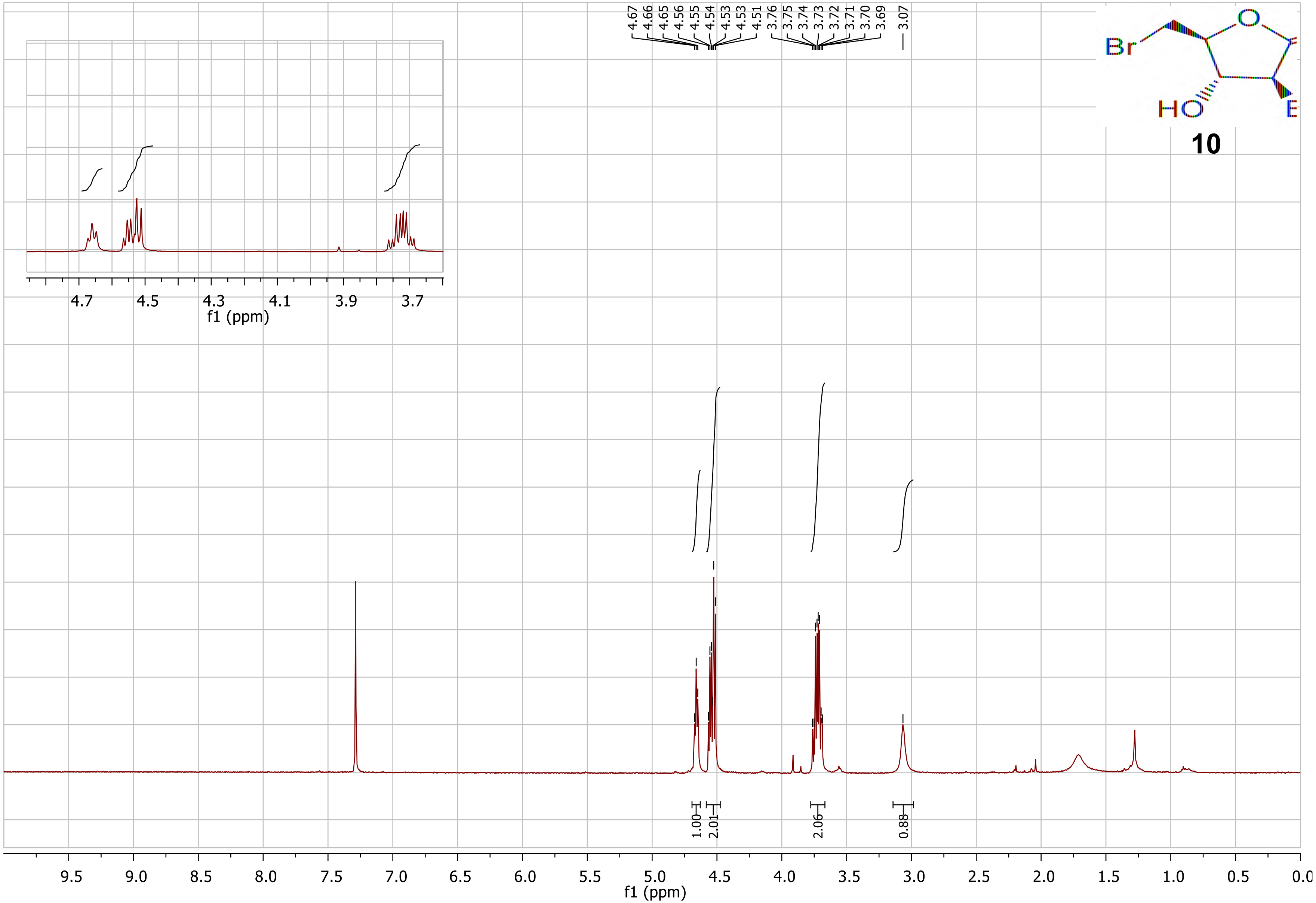

Figure S14: ^{1}H NMR spectrum of compound **10** ($CDCl_3$, 500 MHz).

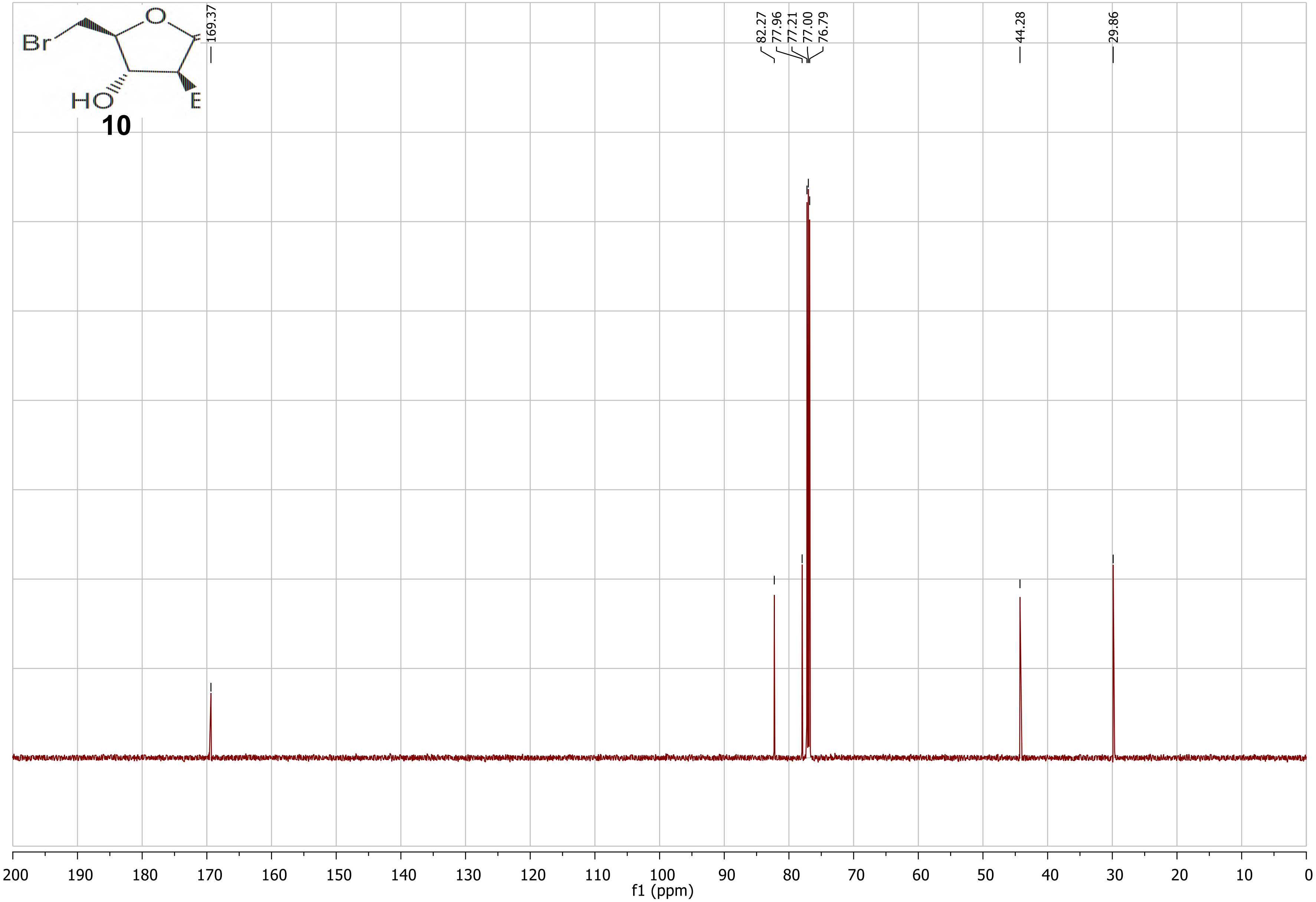

Figure S15: ^{13}C NMR spectrum of compound **10** ($CDCl_3$, 150 MHz).

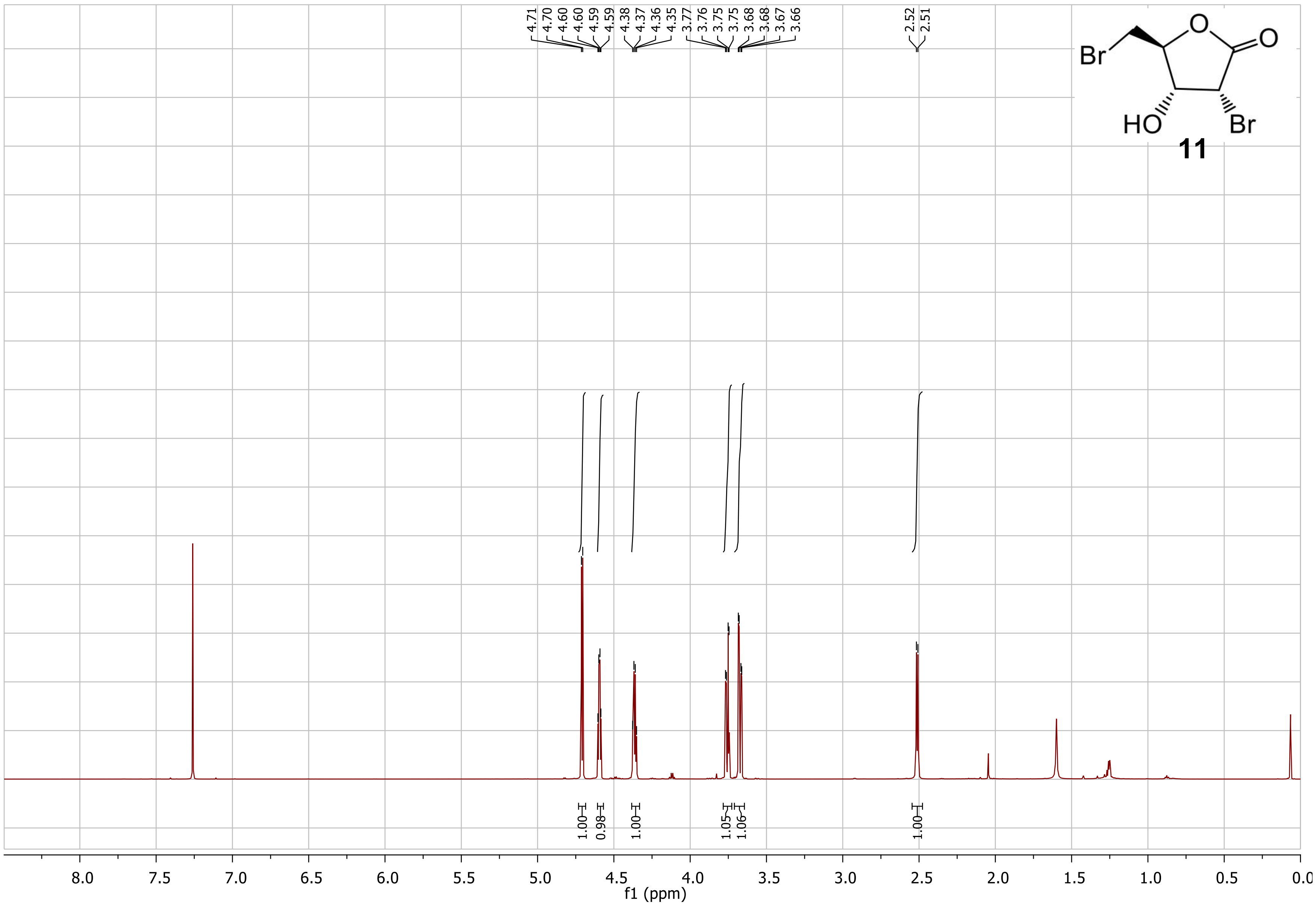

Figure S16: ^{1}H NMR spectrum of compound **11** ($CDCl_3$, 700 MHz).

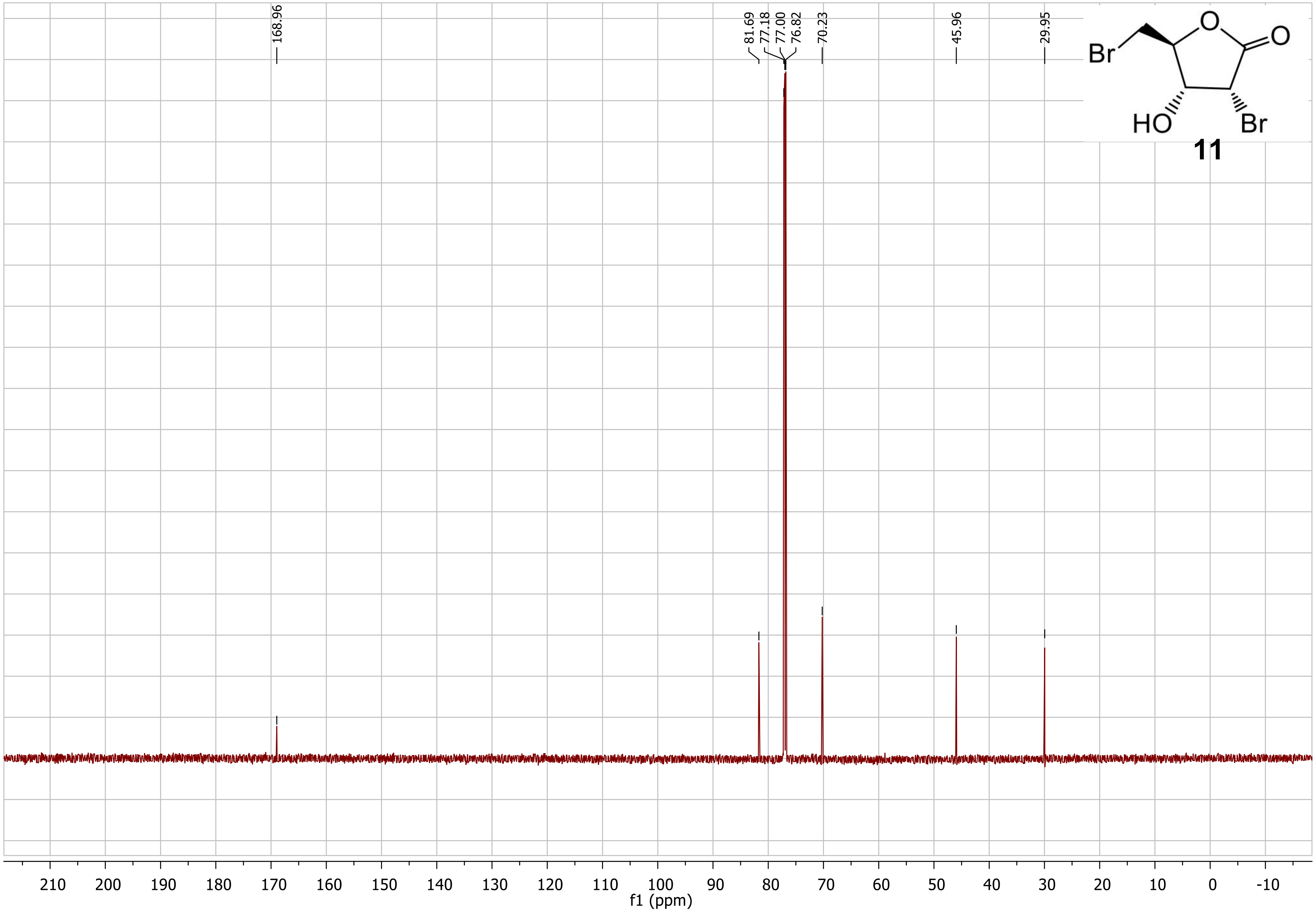

Figure S17: ^{13}C NMR spectrum of compound **11** ($CDCl_3$, 175 MHz).

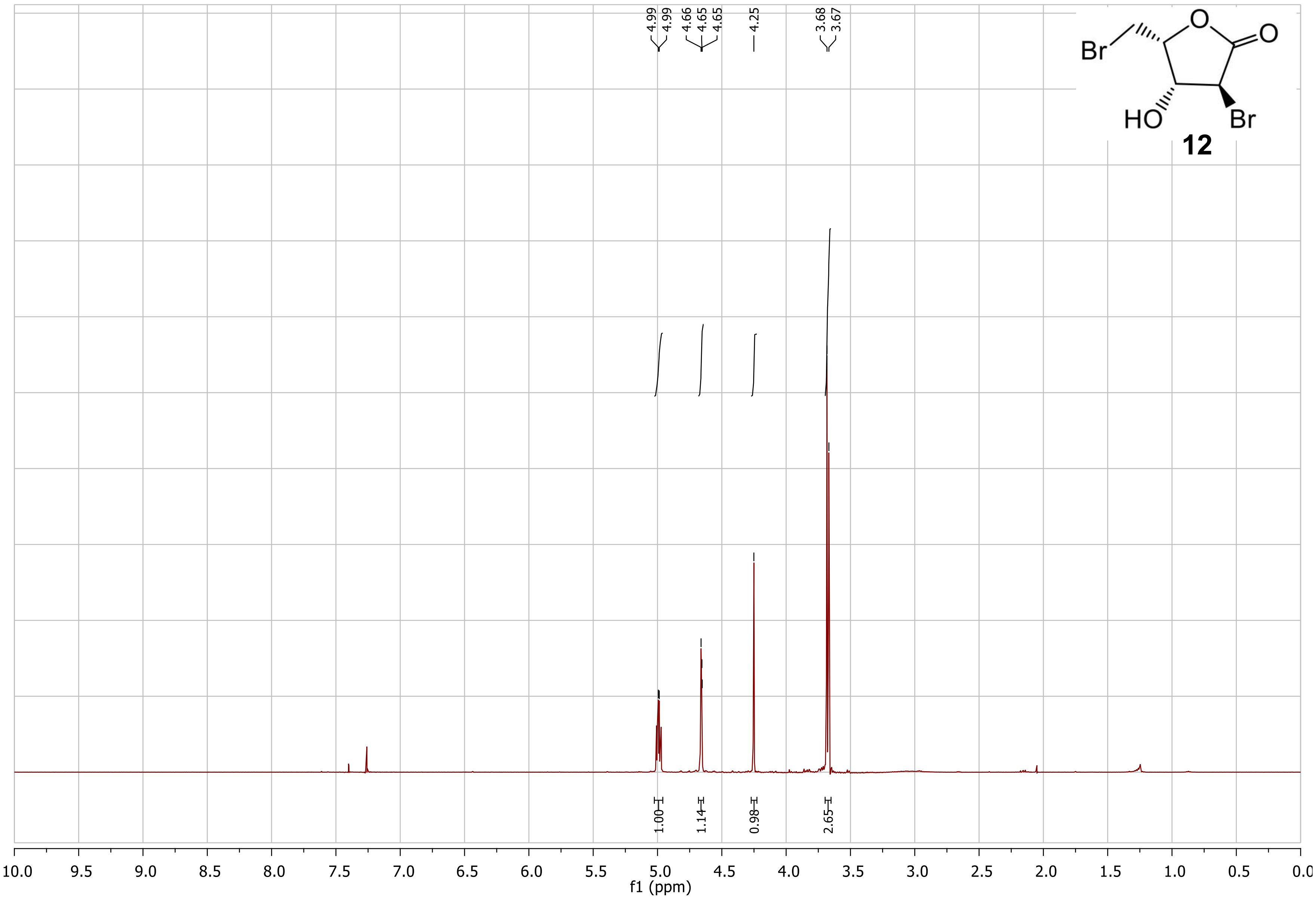

Figure S18: ^{1}H NMR spectrum of compound **12** ($CDCl_3$, 500 MHz).

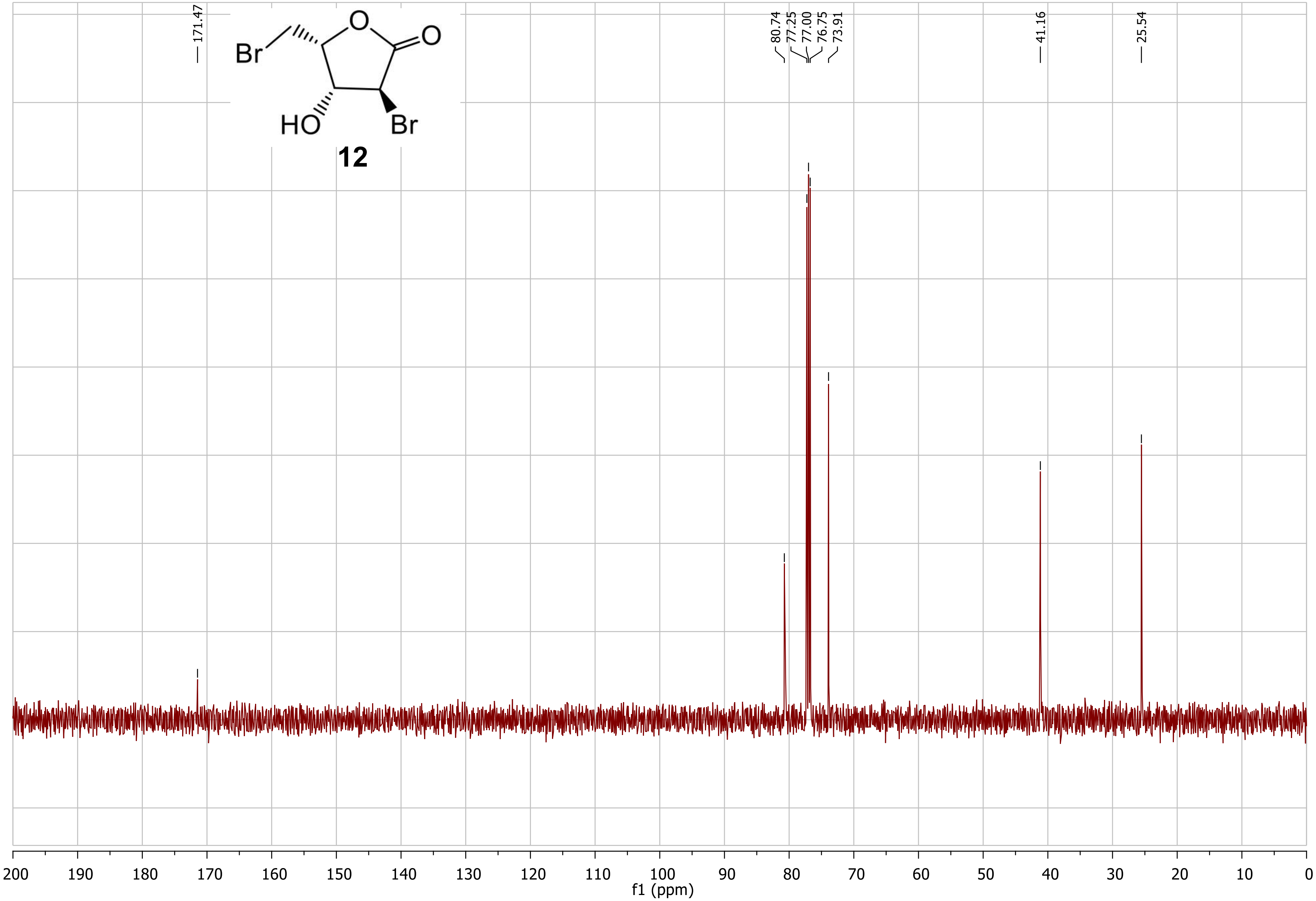

Figure S19: ^{13}C NMR spectrum of compound **12** ($CDCl_3$, 125 MHz).

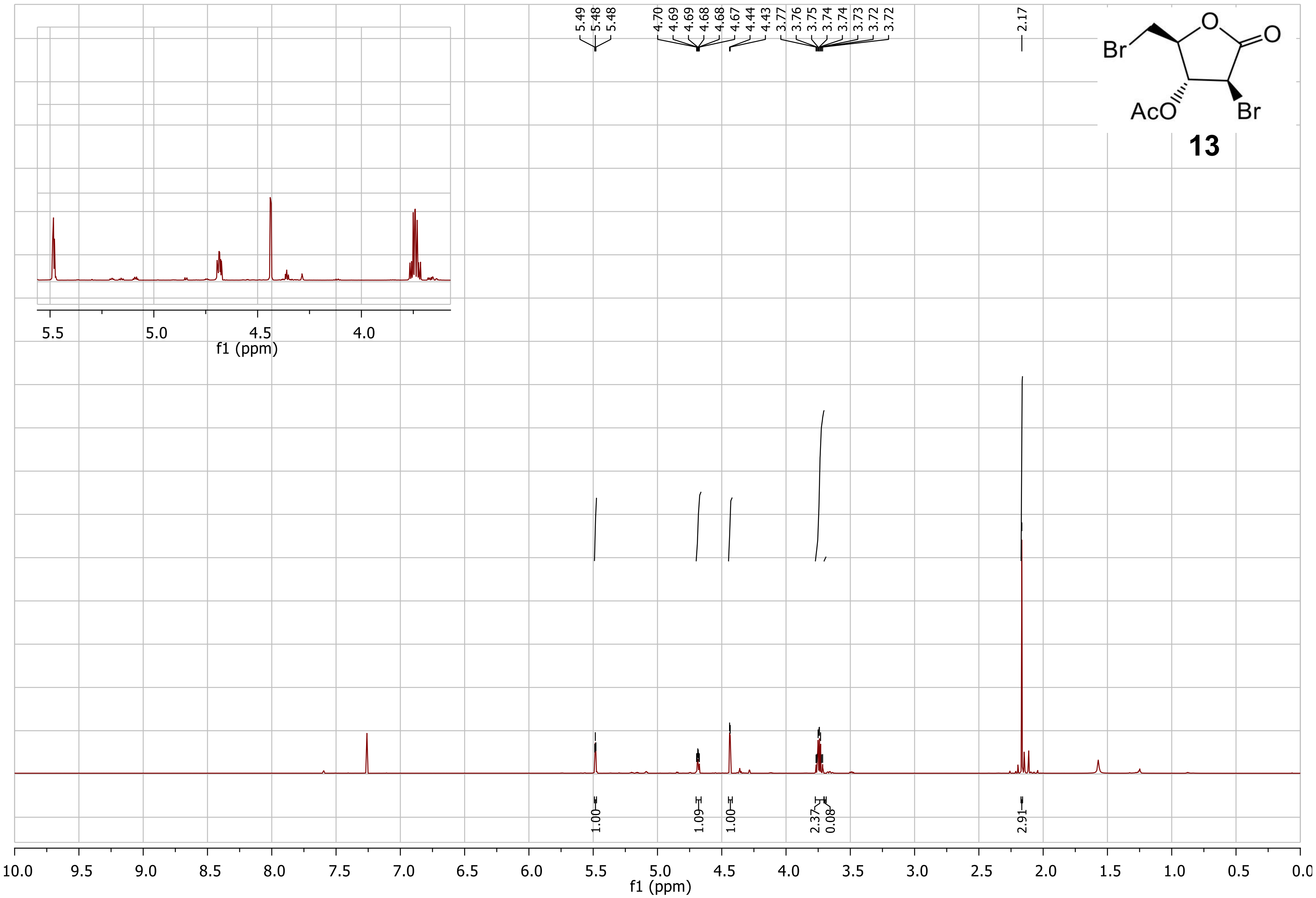

Figure S20: ^{1}H NMR spectrum of compound **13** ($CDCl_3$, 700 MHz).

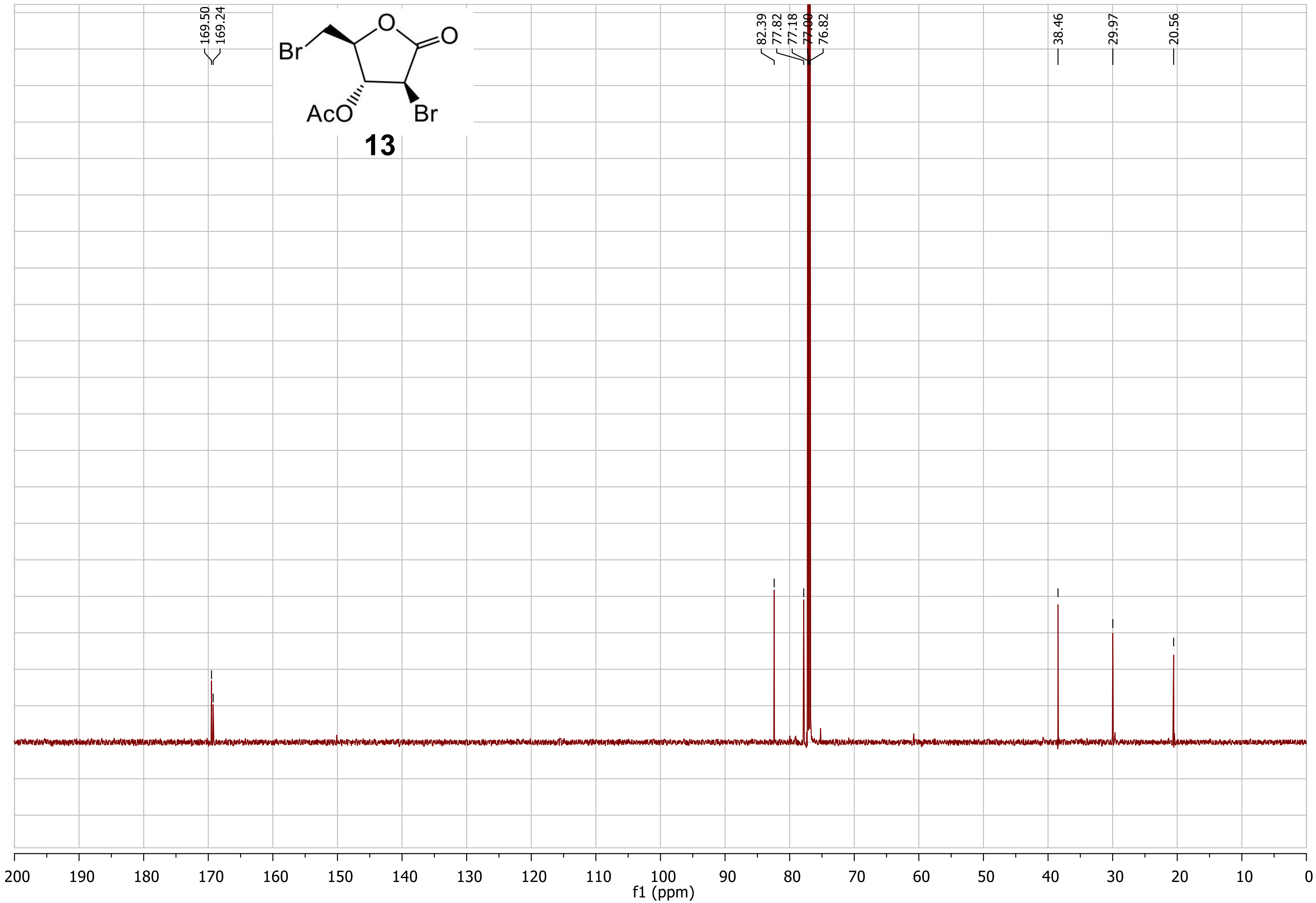

Figure S21 ^{13}C NMR spectrum of compound **13** ($CDCl_3$, 175 MHz).

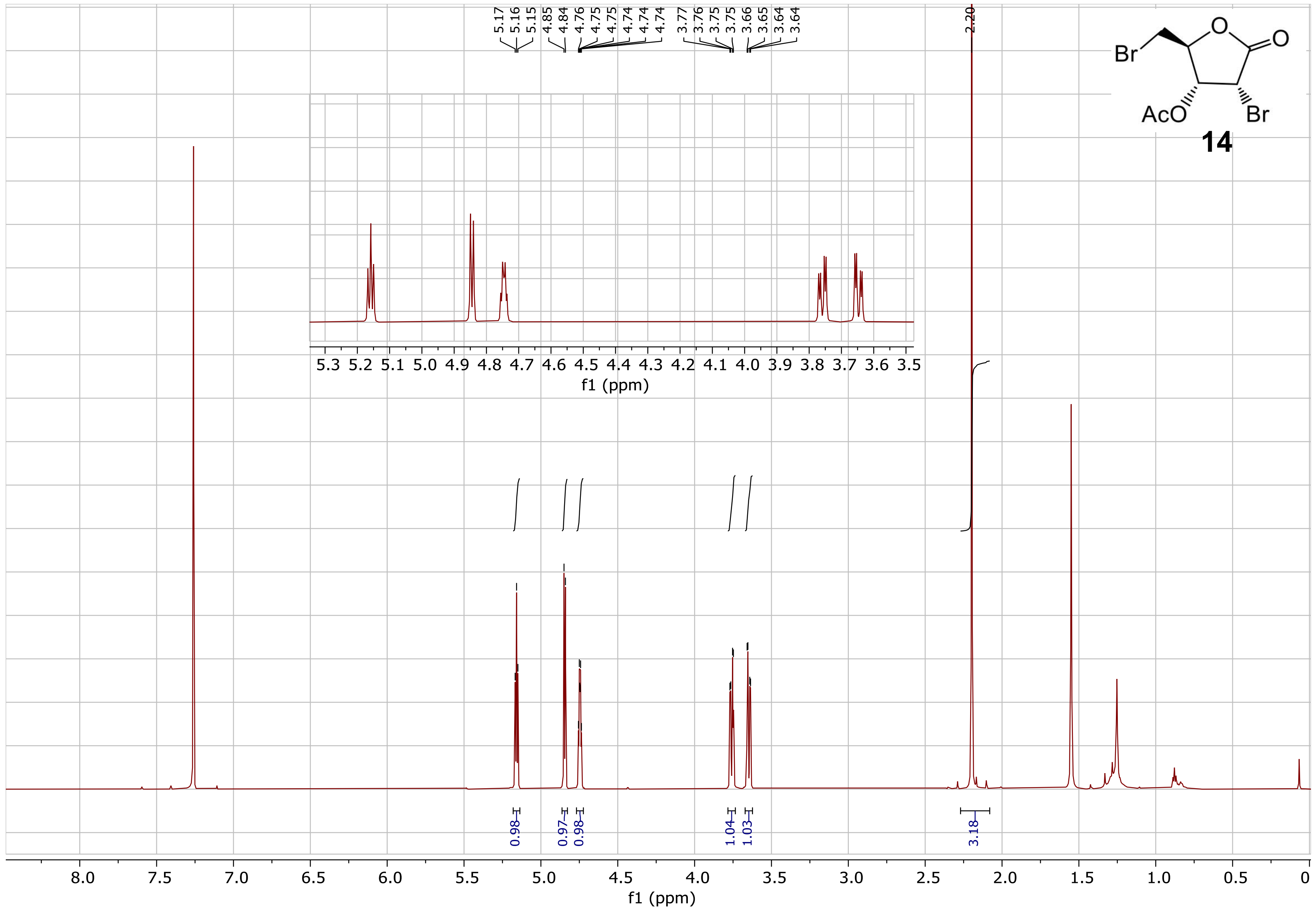

Figure S22: ^{1}H NMR spectrum of **14** ($CDCl_3$, 700 MHz).

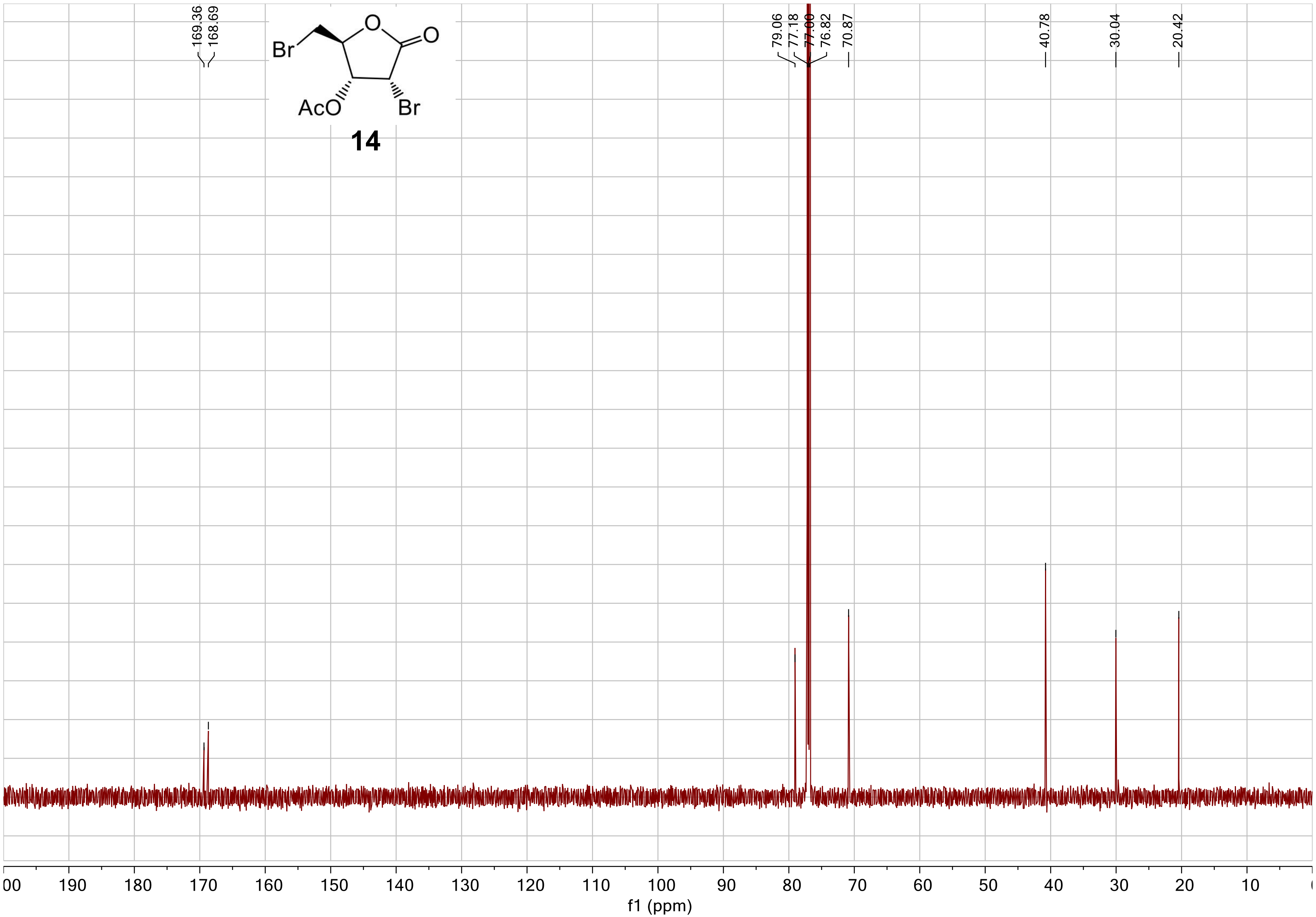

Figure S23: ^{13}C NMR spectrum of compound **14** ($CDCl_3$, 175 MHz).

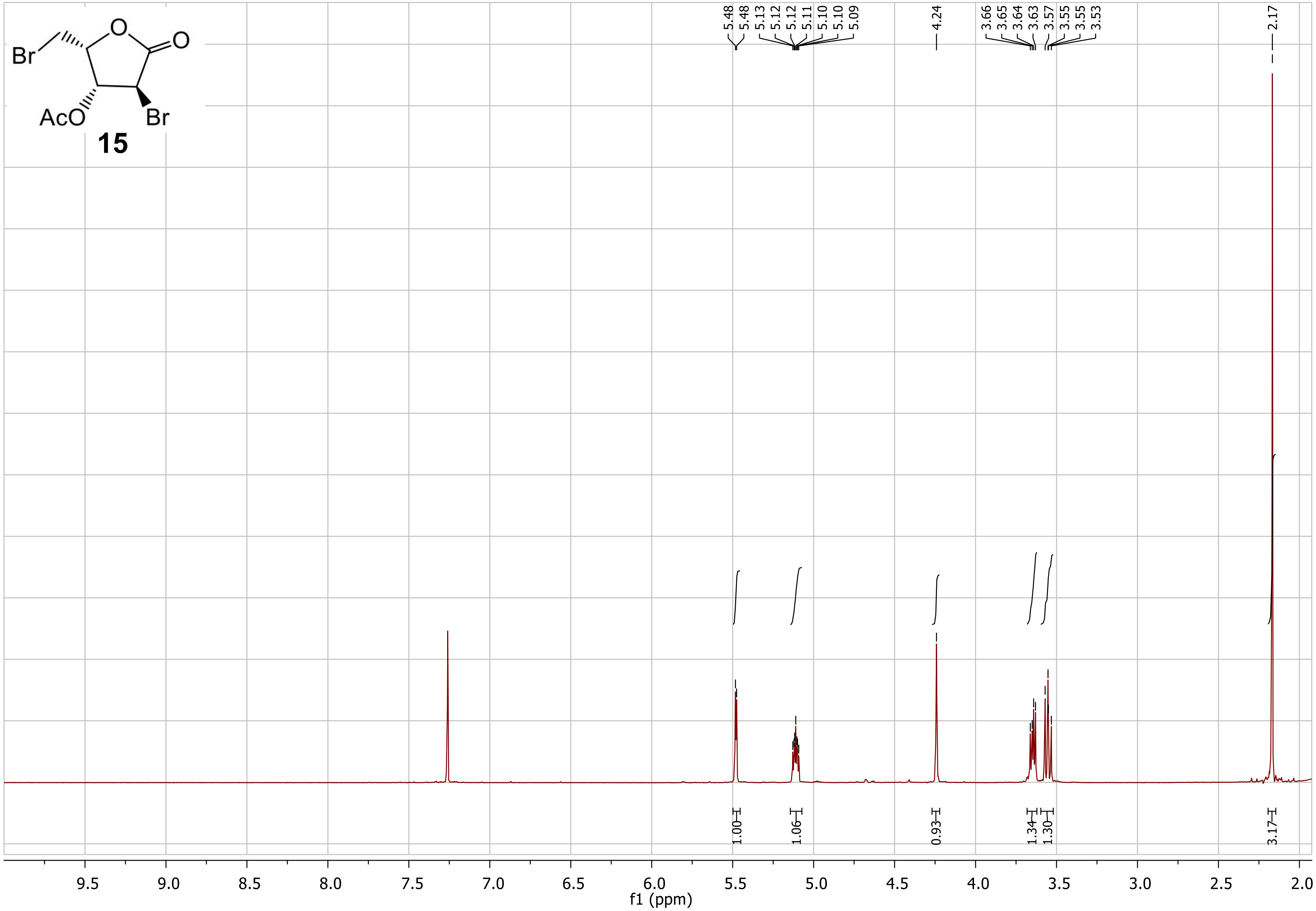

Figure S24: ^{1}H NMR spectrum of **15** ($CDCl_3$, 500 MHz).

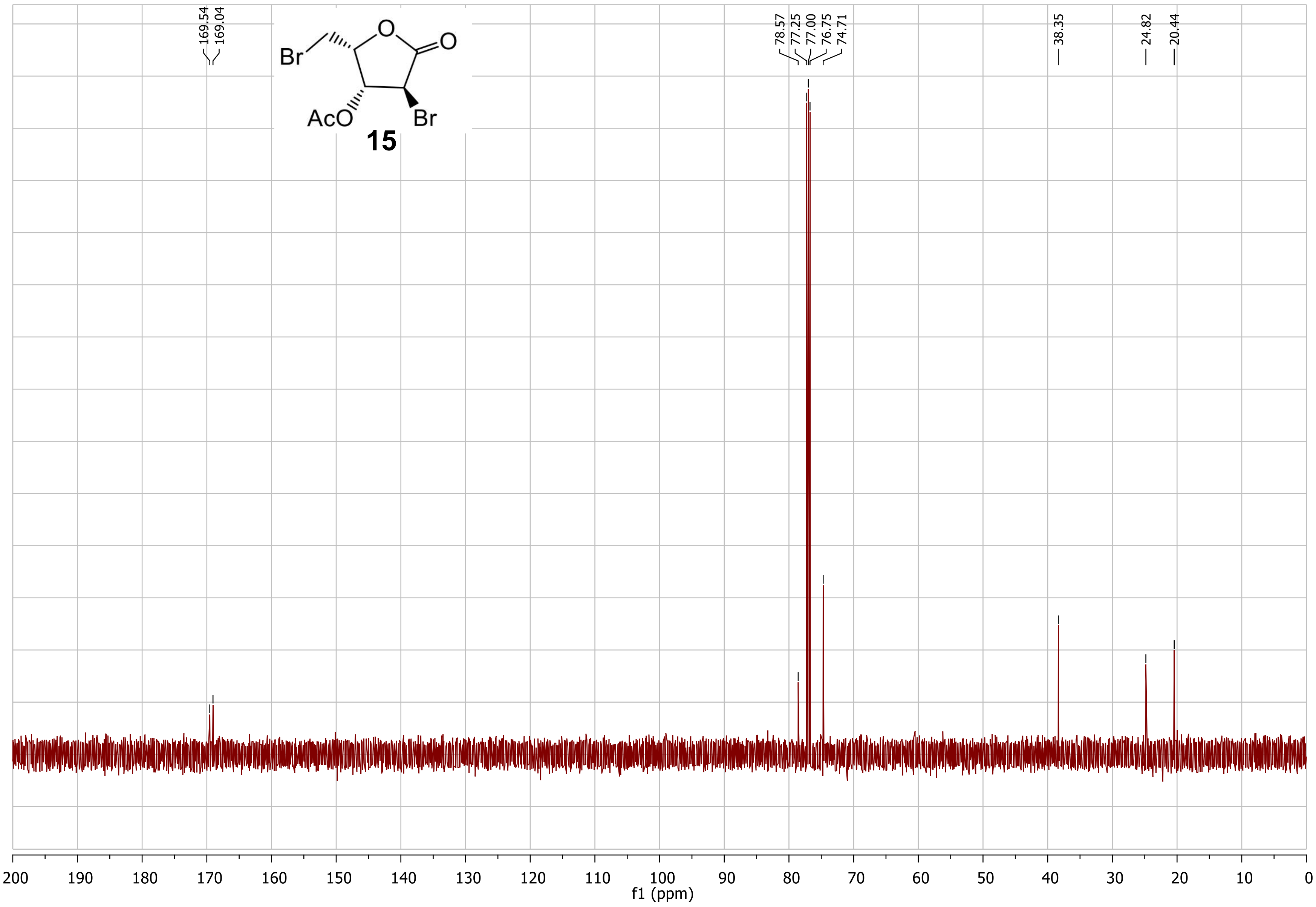

Figure S25: ^{1}H NMR spectrum of **15** ($CDCl_3$, 125 MHz).

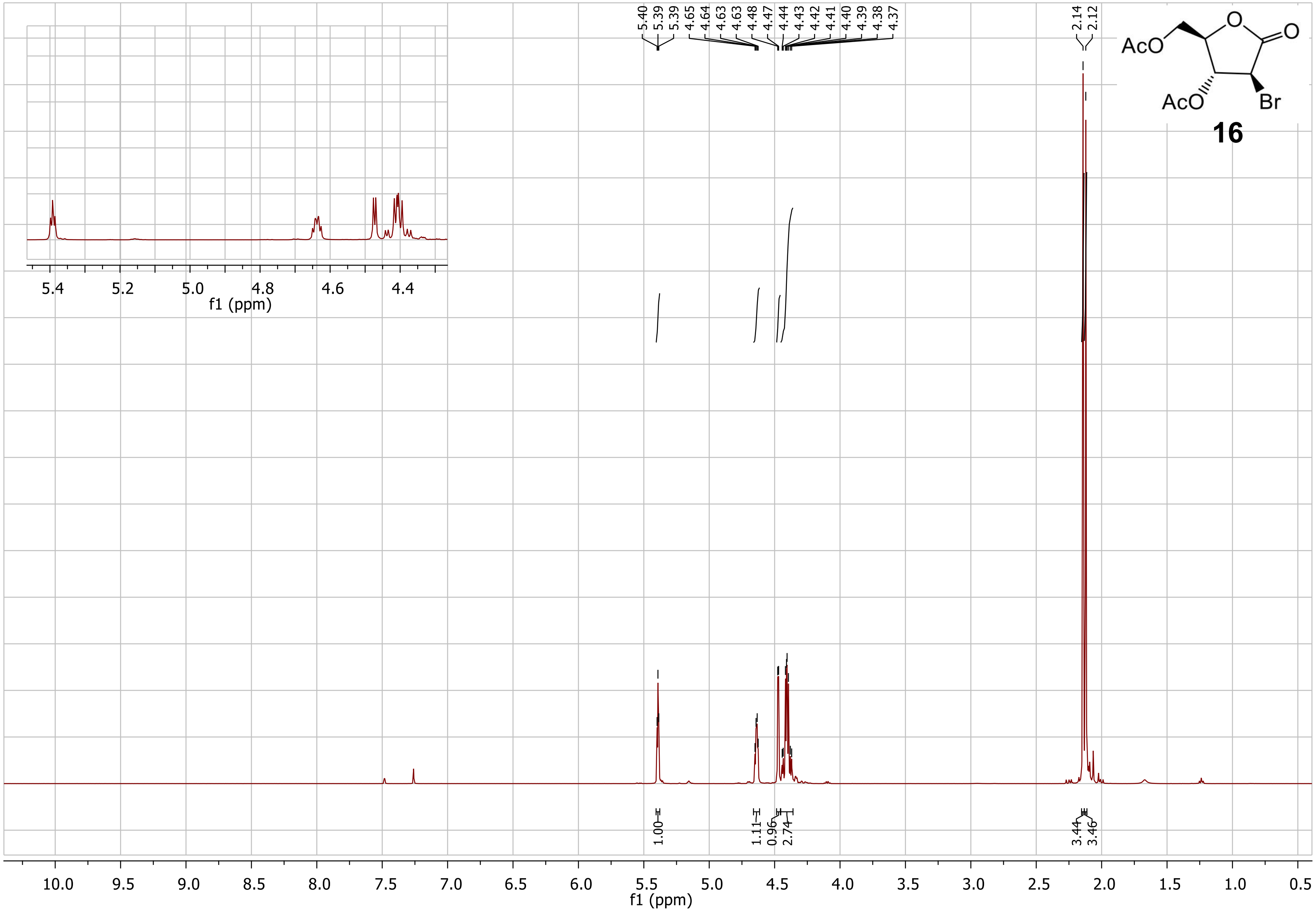

Figure S26: ^{1}H NMR spectrum of **16** ($CDCl_3$, 500 MHz).

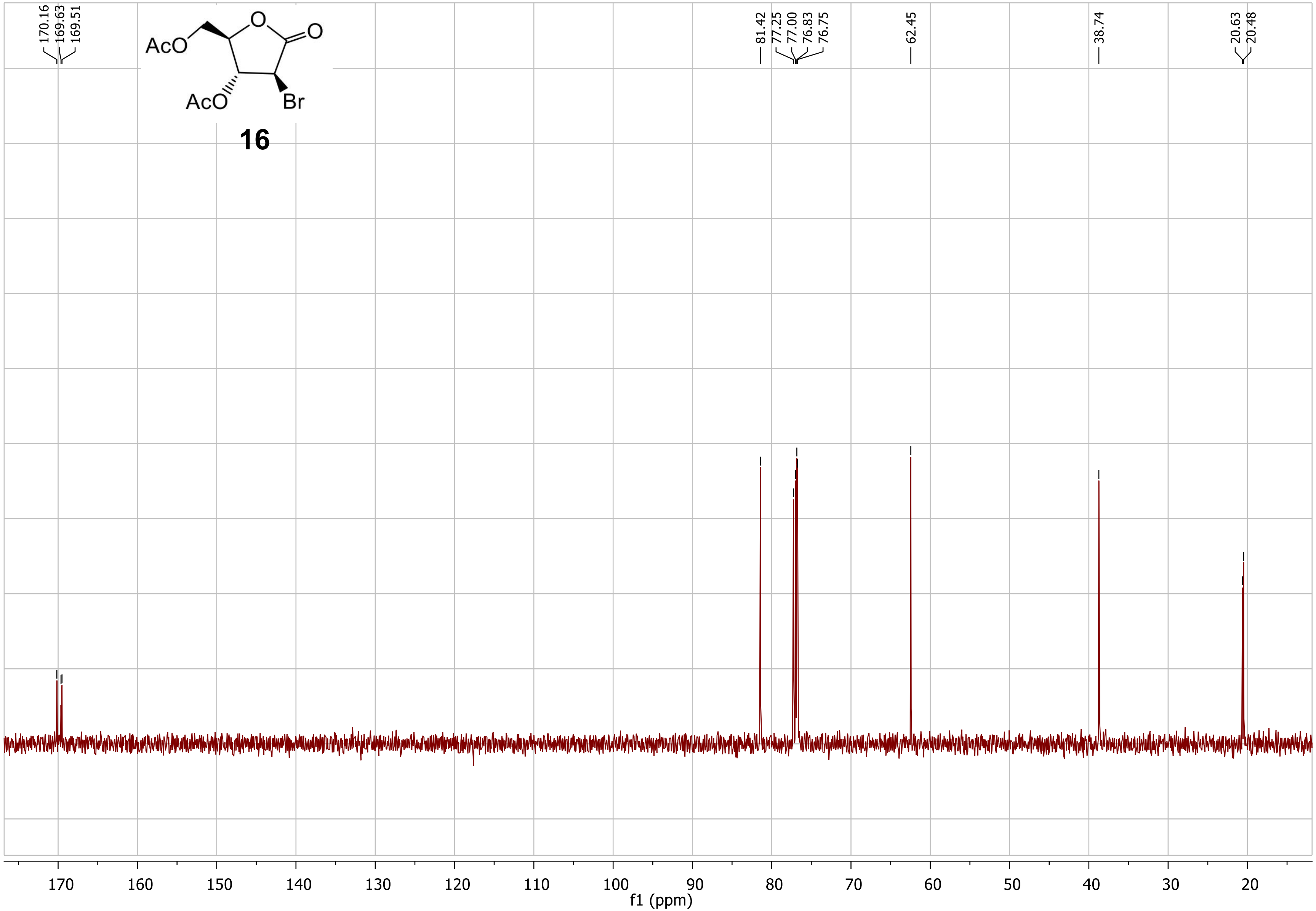

Figure S27: ^{1}H NMR spectrum of **16** ($CDCl_3$, 125 MHz).

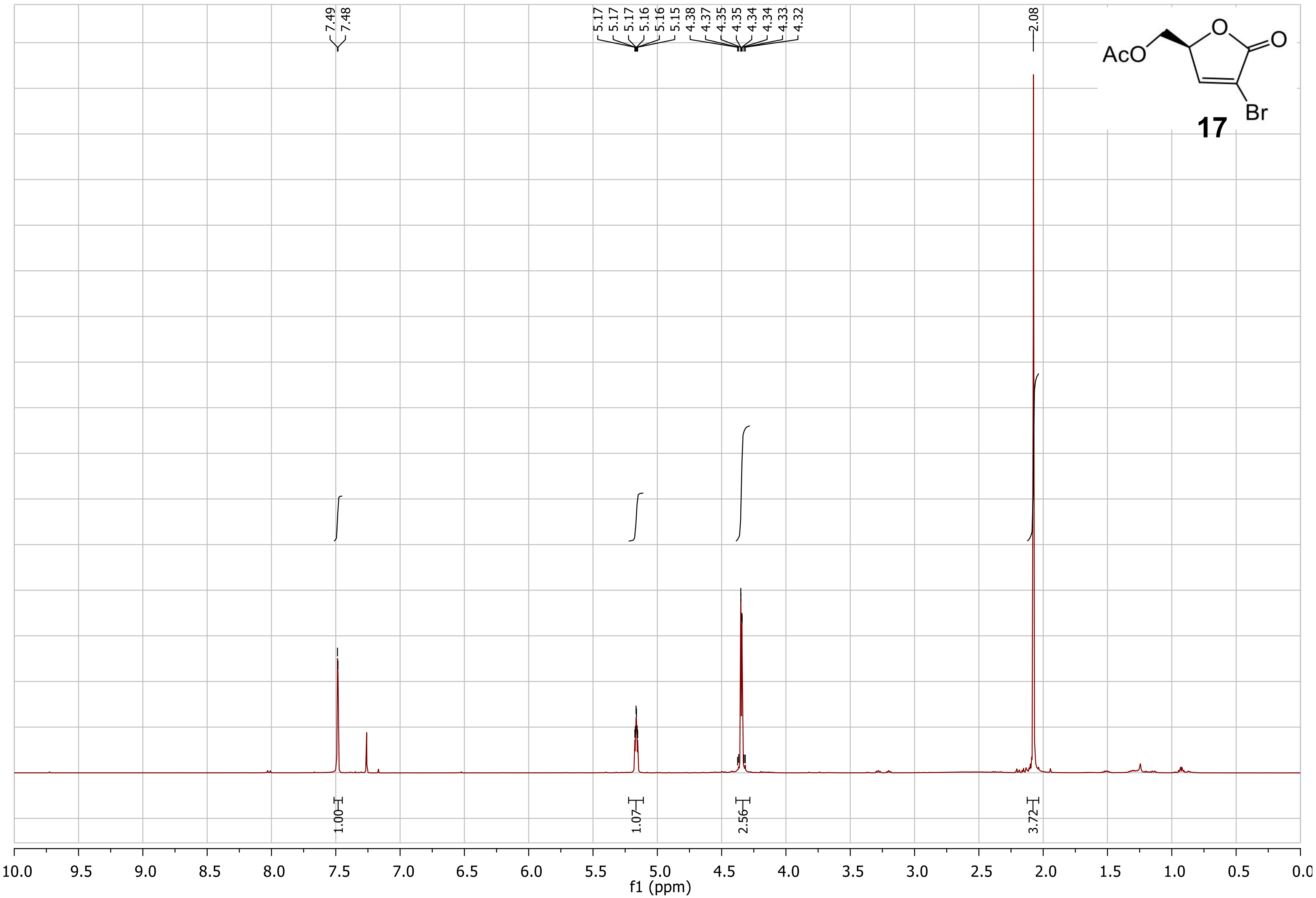

Figure S28: ^{1}H NMR spectrum of compound **17** ($CDCl_3$, 500 MHz).

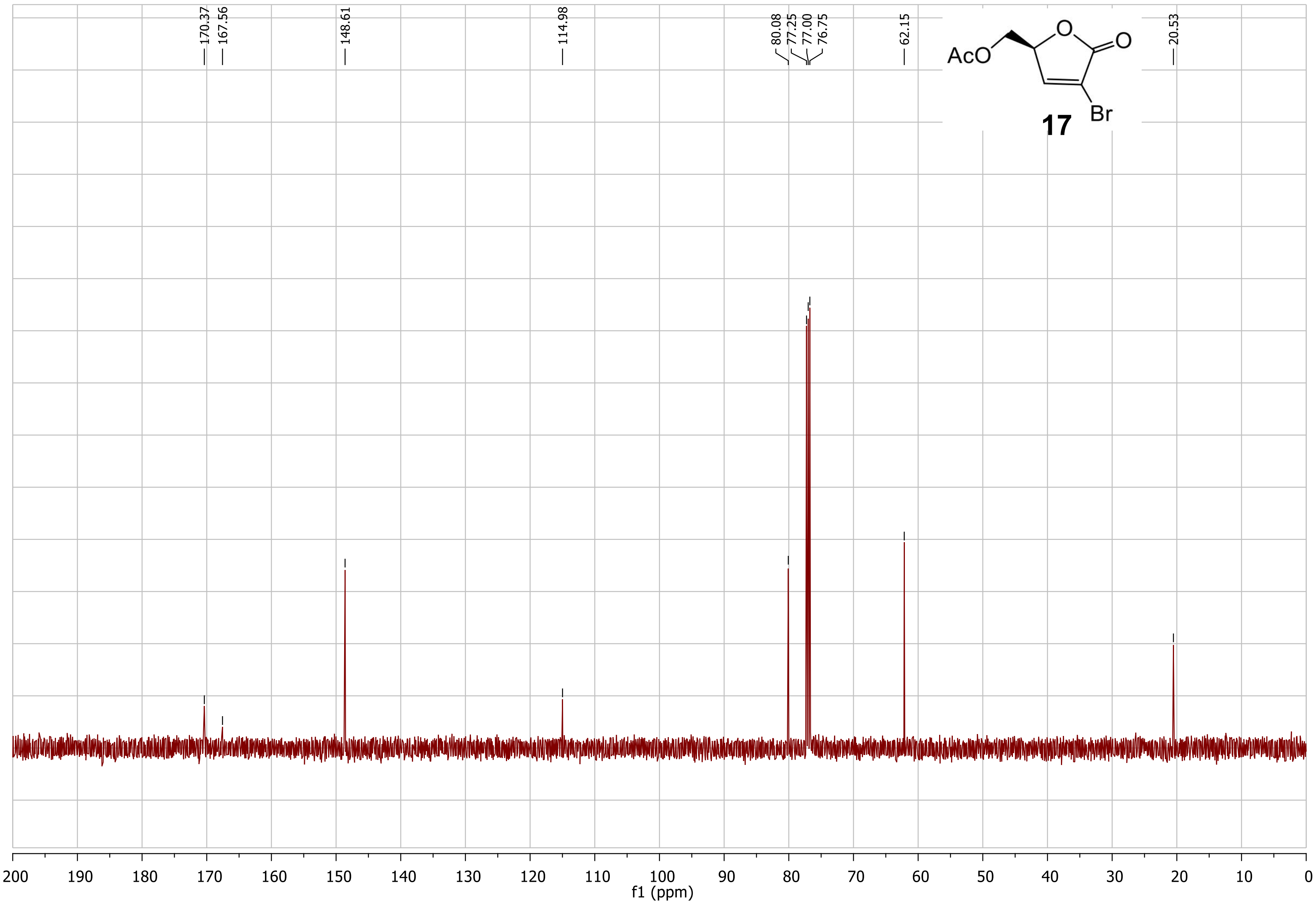

Figure S29: ^{13}C NMR spectrum of compound **17** ($CDCl_3$, 125 MHz).

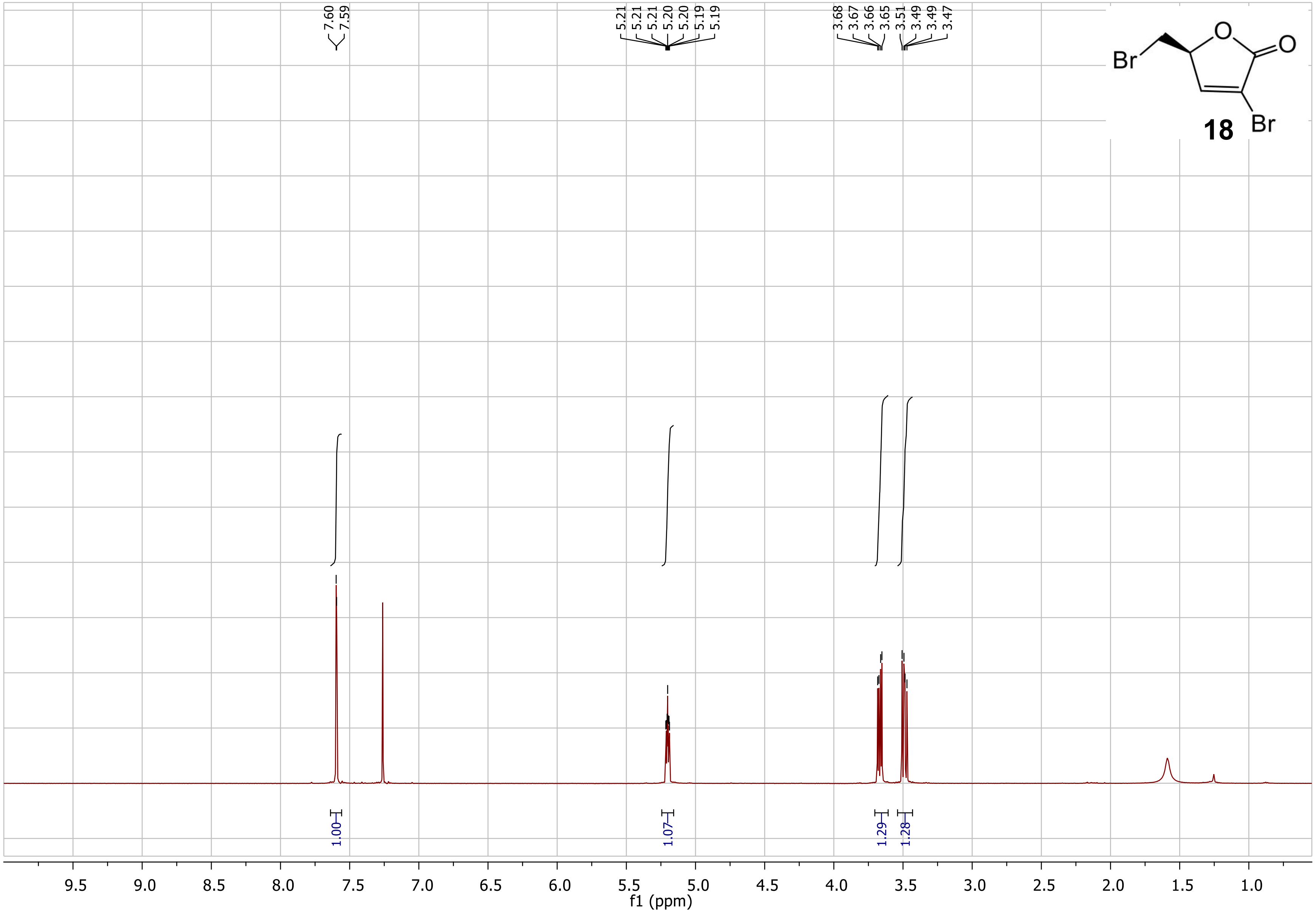

Figure S30: ^{1}H NMR spectrum of compound **18** ($CDCl_3$, 500 MHz).

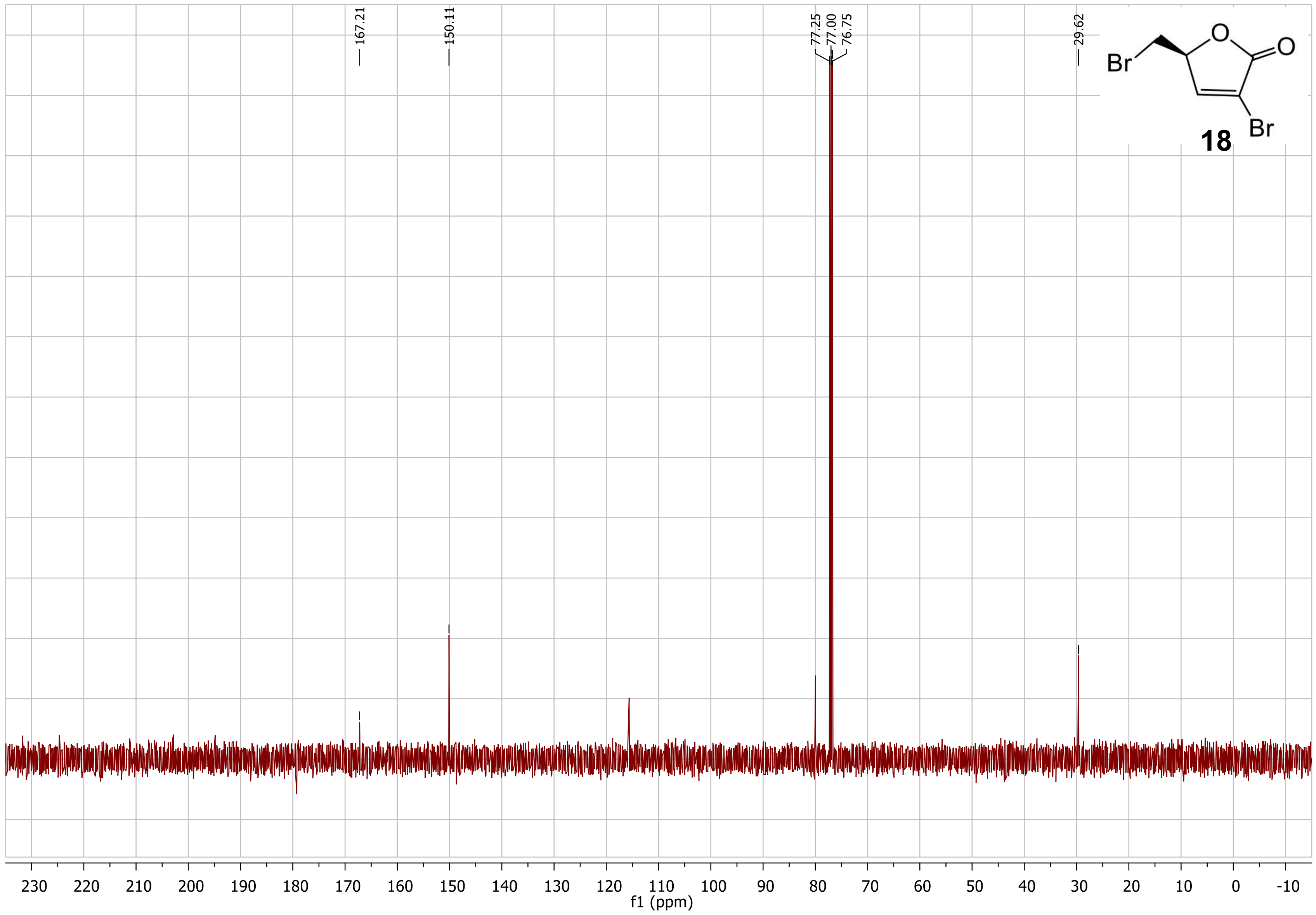

Figure S31: ^{13}C NMR spectrum of compound **18** ($CDCl_3$, 125 MHz).

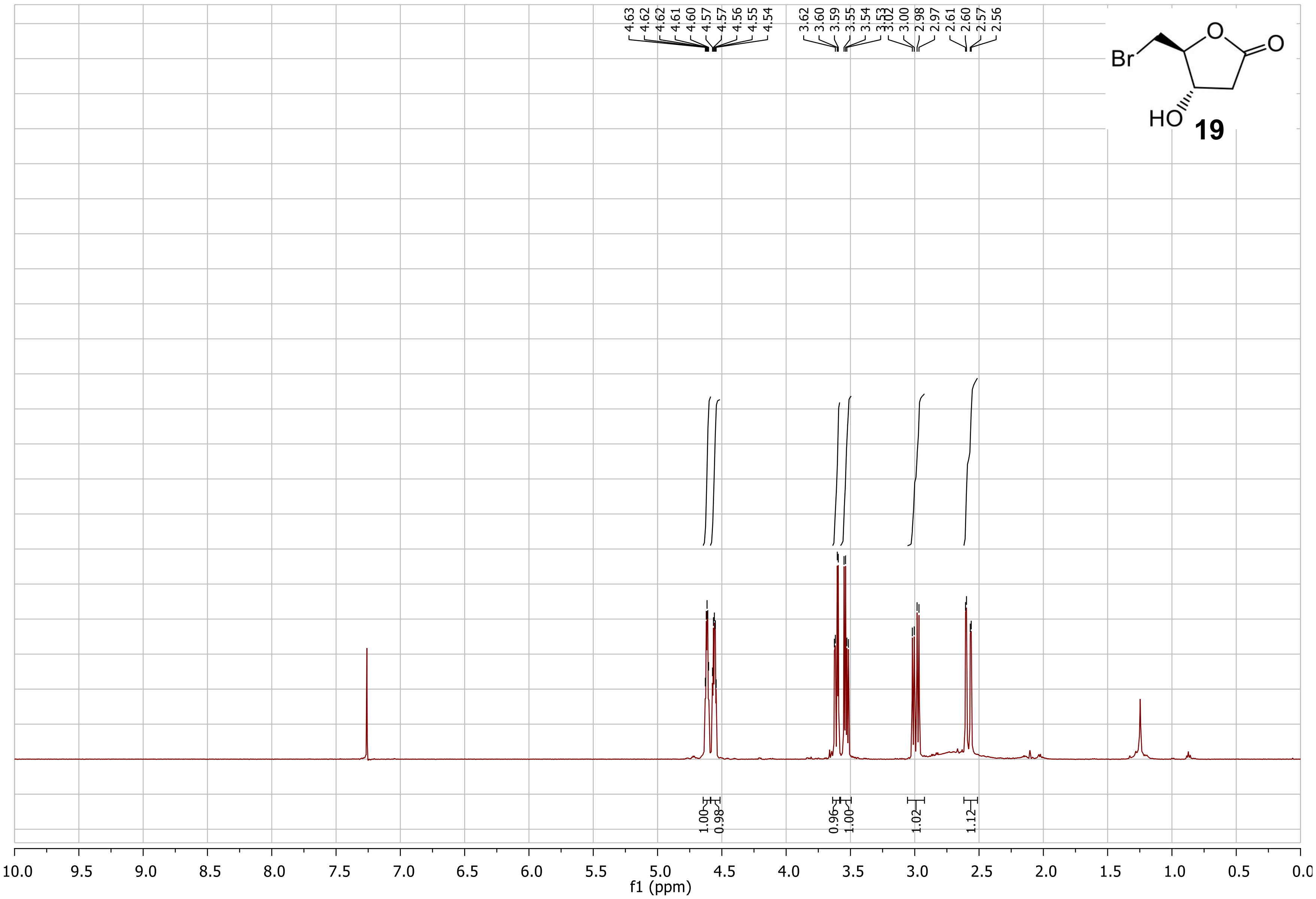

Figure S32: ^{1}H NMR spectrum of compound **19** ($CDCl_3$, 500 MHz).

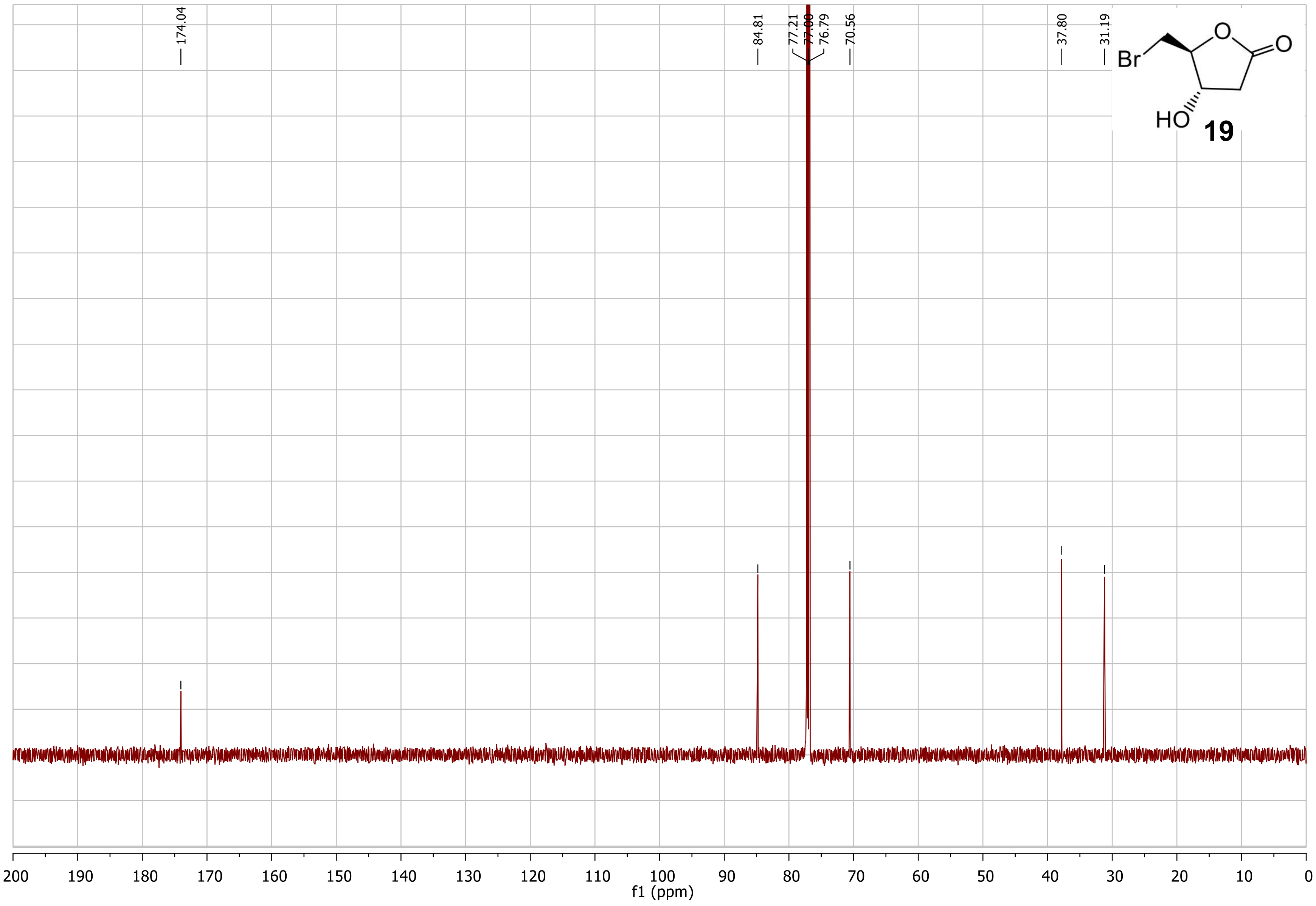

Figure S33: ^{13}C NMR spectrum of compound **19** ($CDCl_3$, 150 MHz).

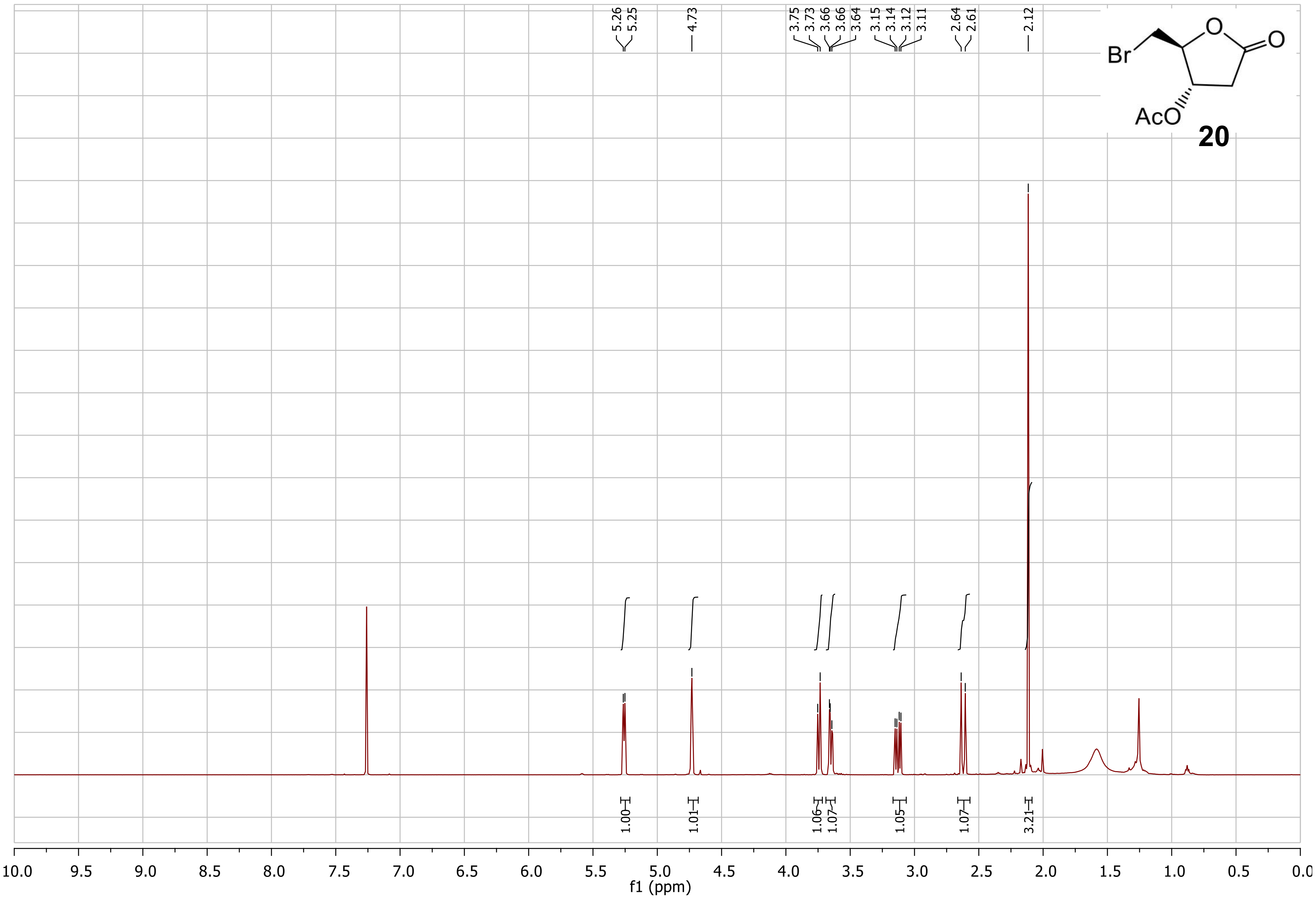

Figure S34: ^{1}H NMR spectrum of compound **20** ($CDCl_3$, 600 MHz).

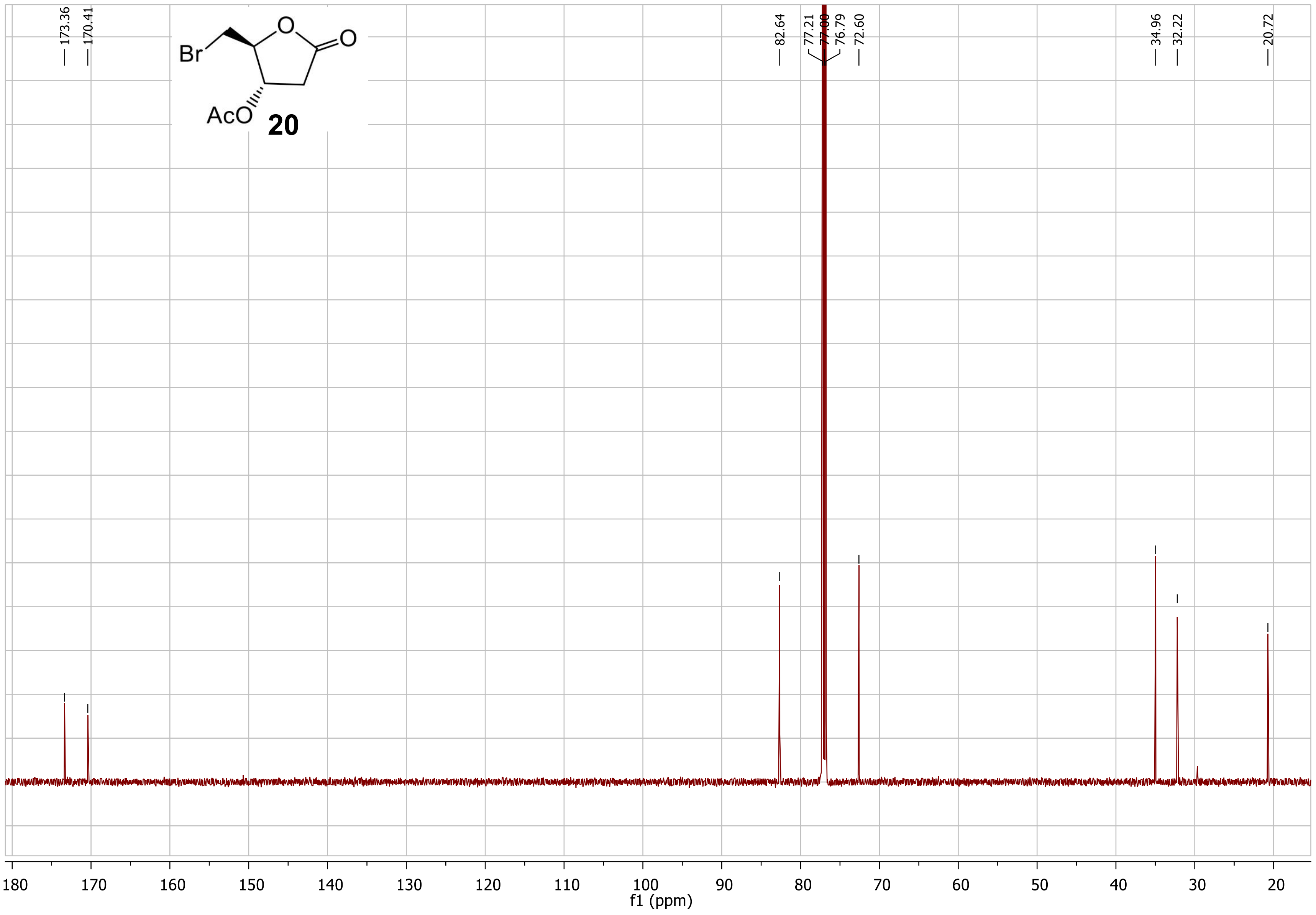

Figure S35: ^{13}C NMR spectrum of compound **20** ($CDCl_3$, 150 MHz).

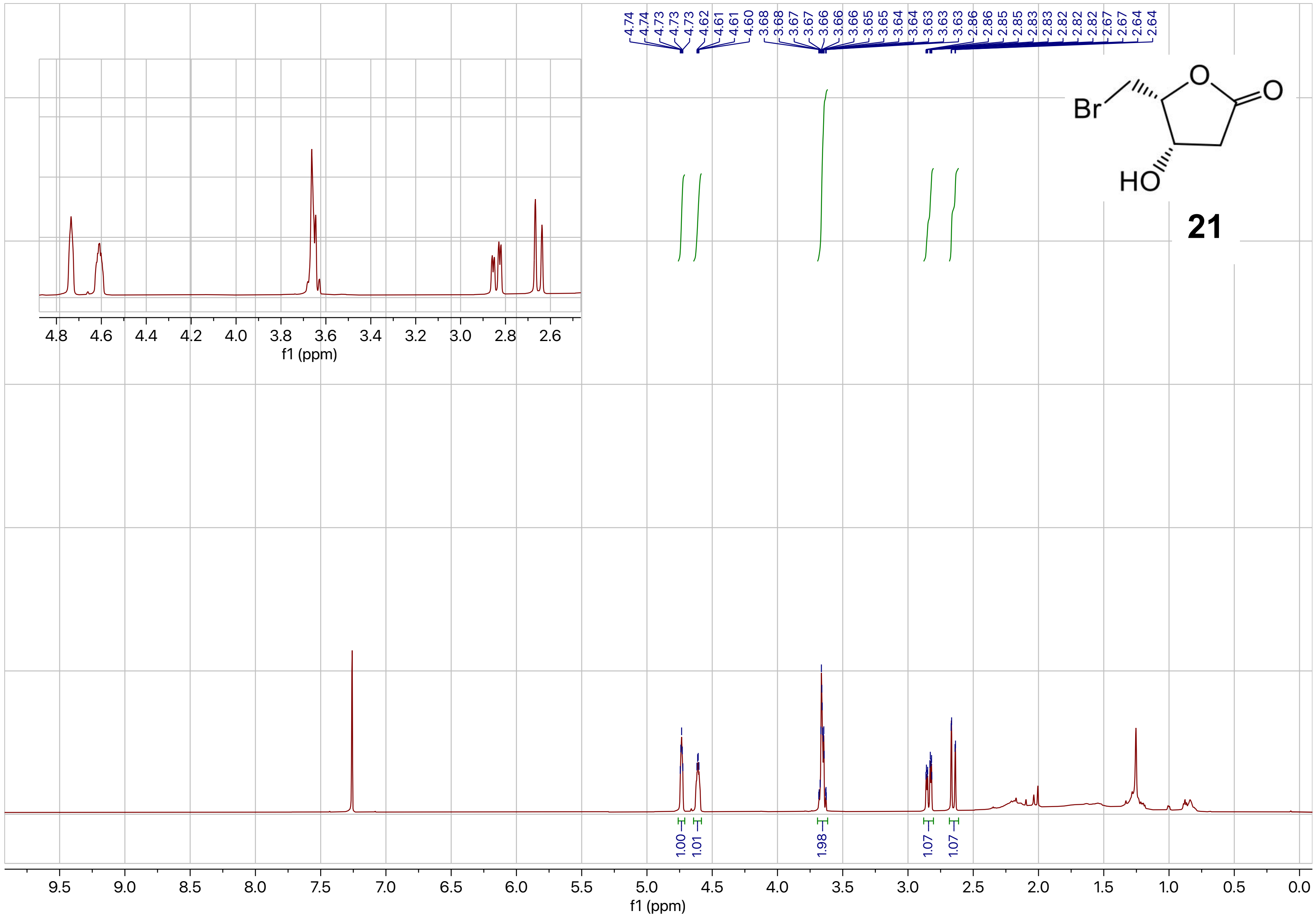

21

Figure S36: ^{1}H NMR spectrum of compound **21** ($CDCl_3$, 700 MHz).

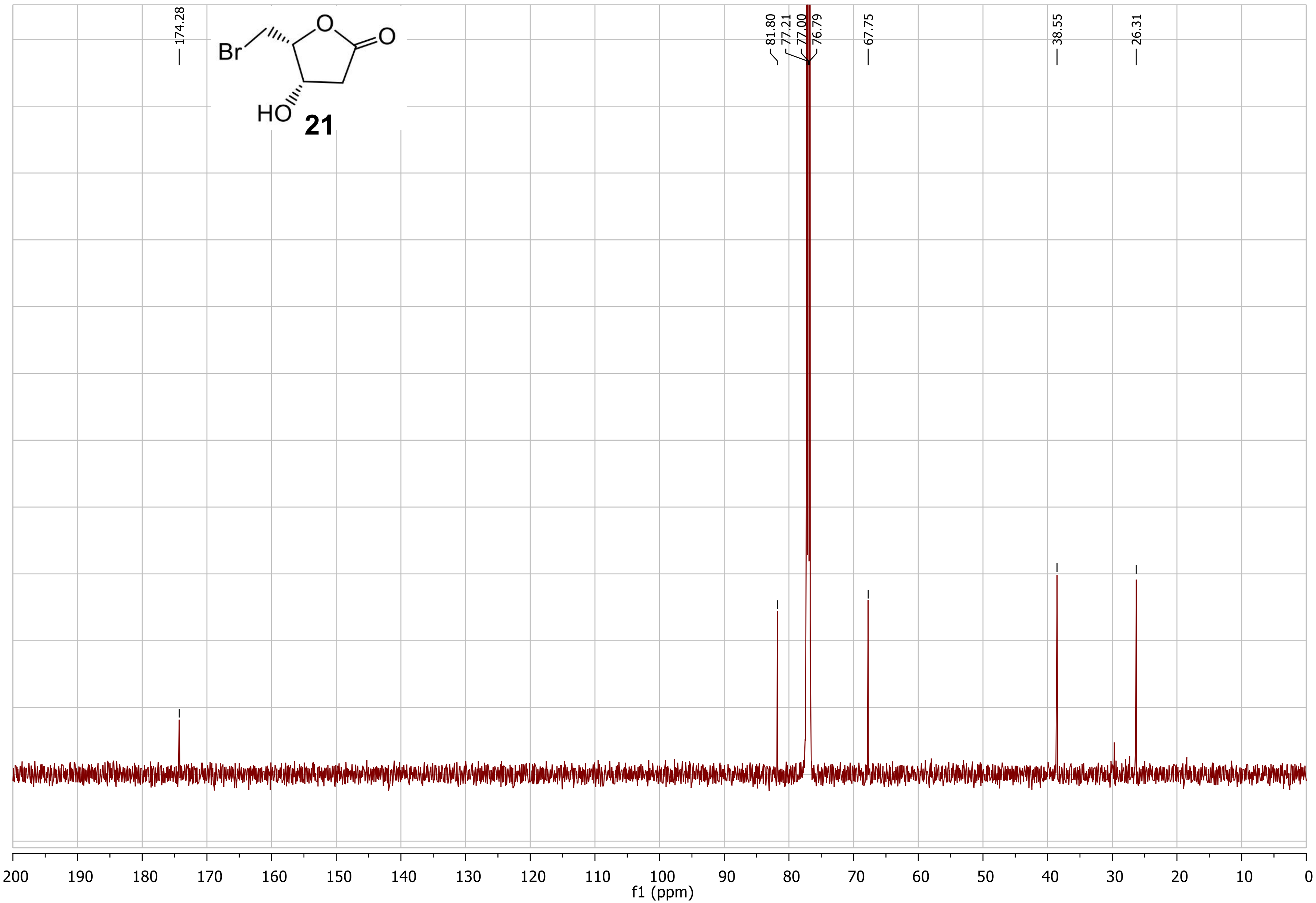

Figure S37: ^{13}C NMR spectrum of compound **21** ($CDCl_3$, 175 MHz).

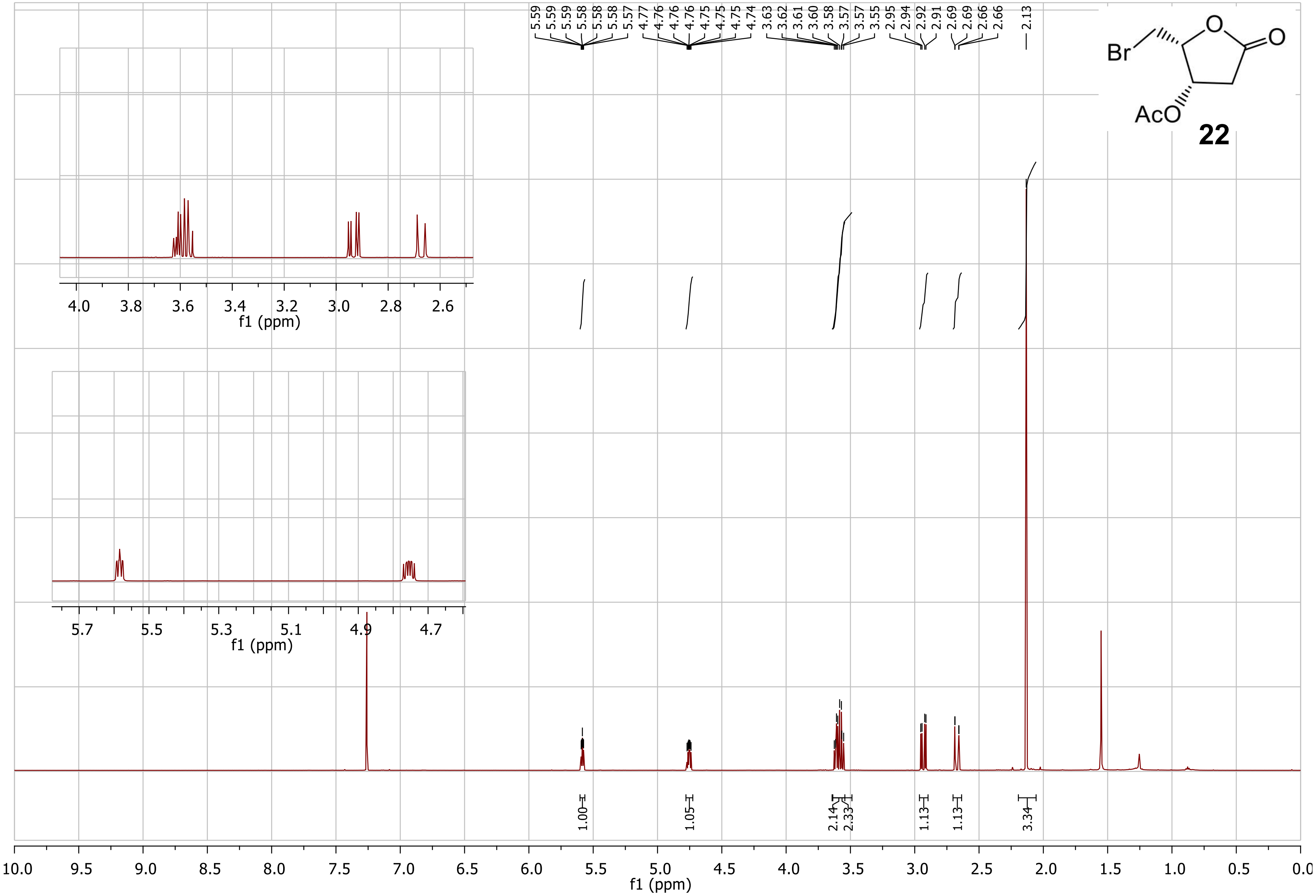

Figure S38: ^{1}H NMR spectrum of compound **22** ($CDCl_3$, 700 MHz).

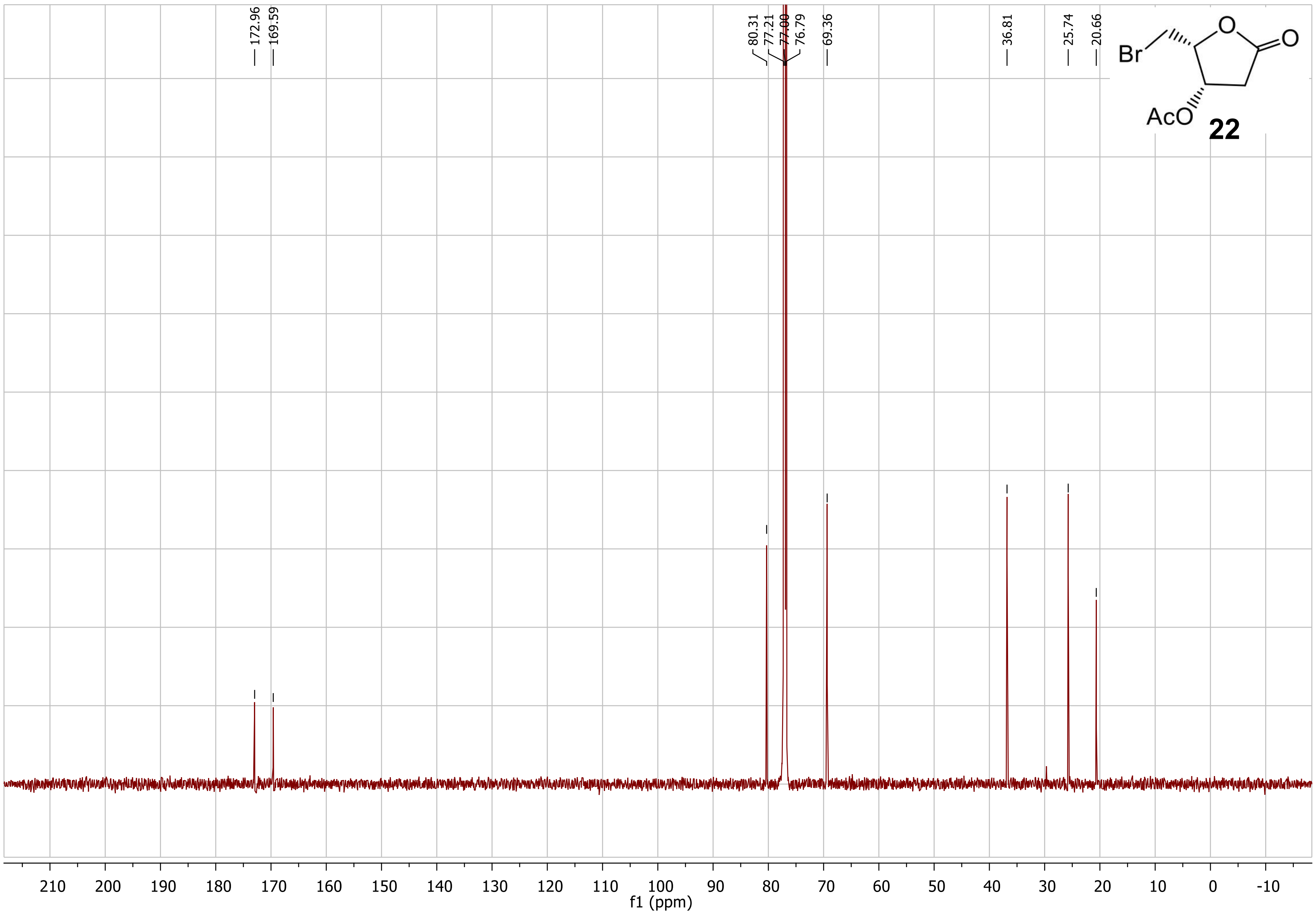

Figure S39: ^{13}C NMR spectrum of compound **22** ($CDCl_3$, 175 MHz).
